# The Structure of Monomeric Hydroxo-Cu^II^ Species in Cu-CHA. A Quantitative Assessment

**DOI:** 10.1021/jacs.2c06037

**Published:** 2022-07-12

**Authors:** Paolo Cleto Bruzzese, Enrico Salvadori, Bartolomeo Civalleri, Stefan Jäger, Martin Hartmann, Andreas Pöppl, Mario Chiesa

**Affiliations:** † Felix Bloch Institute for Solid State Physics, Leipzig University, 04103 Leipzig, Germany; ‡ Department of Chemistry and NIS Centre of Excellence, University of Turin, 10125 Torino, Italy; § Erlangen Center for Interface Research and Catalysis (ECRC), FAU Erlangen-Nürnberg, 91058 Erlangen, Germany

## Abstract

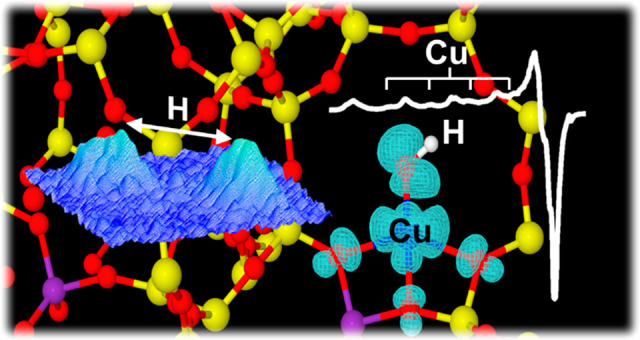

Using EPR and HYSCORE spectroscopies in conjunction with *ab initio* calculations, we assess the structure of framework-bound monomeric hydroxo-Cu^II^ in copper-loaded chabazite (CHA). The species is an interfacial distorted square-planar [Cu^II^OH(O-8MRs)_3_] complex located at eight-membered-ring windows, displaying three coordinating bonds with zeolite lattice oxygens and the hydroxo ligand hydrogen-bonded to the cage. The complex has a distinctive EPR signature with **g** = [2.072 2.072 2.290], ^Cu^**A**= [30 30 410] MHz, and ^H^**A** = [−13.0 −4.5 +11.5] MHz, distinctively different from other Cu^II^ species in CHA.

Copper-exchanged chabazite (CHA) zeolites, a class of materials promoting a wide range of important catalytic transformations,^1^ feature a plurality of different copper species ranging from monomeric copper sites^2^ to dinuclear ([Cu_2_O]^2+^ and [Cu_2_O_2_]^2+^)^3,4^ and polynuclear clusters,^5^ each one related to specific catalytic functions.^6−8^

These different copper species are crucially related to the zeolite composition and exhibit unique spectroscopic features, which reflect specific electronic structures that can make key contributions to reactivity. For example, specific (μ-oxo)dicopper(II) species ([Cu_2_O]^2+^) in Cu-CHA have been recently shown to react with methane to form methanol at low temperature.^3^

The precursors of such copper-coupled dimers are paramagnetic monocopper hydroxo species ([Cu(OH)]^+^).^8,9^ Despite their relevance, however, geometric and electronic structure/function correlations are scanty.

Recent studies^10−14^ indicate the presence of two major Cu sites in activated Cu-CHA, namely, interfacial complexes coordinated with four oxygen donor atoms of the six-membered-ring (6MR) cavities ([Cu^II^(O-6MR)_4_]), whereby Cu^II^ is stabilized by two neighboring charge-balancing Al^3+^ framework ions, and [Cu^II^(OH)]^+^ species at eight-membered-ring (8MR) cages next to an isolated Al^3+^ ion. Importantly, only the second complex is redox-active, allowing for Cu^II^/Cu^I^ redox cycles.^8,9,13^ The relative population of the two species depends on the number and distribution of Al ions in the framework and on the Cu/Al ratio. Materials with low Si/Al and Cu/Al ratios should contain predominantly [Cu^II^(O-6MR)_4_] species, while materials with high Si/Al and Cu/Al ratios are dominated by [Cu^II^(OH)]^+^ species.^15,16^

First suggested by Valyon,^17^ [Cu^II^(OH)]^+^ has been identified in high loaded Cu-CHA (Si/Al = 11–15) after dehydration in O_2_/He by Giordanino and Borfecchia et al.^13,18^ through characteristic IR stretching and bending bands at 3656 and 905 cm^–1^. Electron paramagnetic resonance (EPR) spectroscopy has been used to indirectly evaluate the amount of [Cu^II^(OH)]^+^;^19−22^ however, no direct detection of these species in dehydrated Cu-CHA has been reported so far. Herein we identify the EPR signature of framework-bound [Cu^II^(OH)]^+^ species, elucidating their structure that is different from past assignments.^2,8,13,15,21−23^ The structural requirement for the stabilization of [Cu^II^(OH)]^+^ is based on the availability of single Al framework sites, which are assumed to be populated after 2Al sites.^12^ Paolucci et al.^12^ computed a Cu-site compositional phase diagram for O_2_-activated Cu-CHA, under the assumptions that Al is distributed randomly and the Loewenstein’s rule^24^ is obeyed (i.e., Al–O–Al sites are avoided). On the basis of this diagram (Figure 1a), we chose four different Cu-CHA compositions corresponding to different [Cu^II^(OH)]^+^ predicted populations ranging from approximately 0 (A in Figure 1a) to about 40% of total Cu (point D in Figure 1a). The samples were activated by adopting conditions known to maximize [Cu^II^(OH)]^+^ sites (dehydration at 523 K under an O_2_ atmosphere),^8^ and the corresponding EPR spectra are shown in Figure 1b.

**Figure 1:**
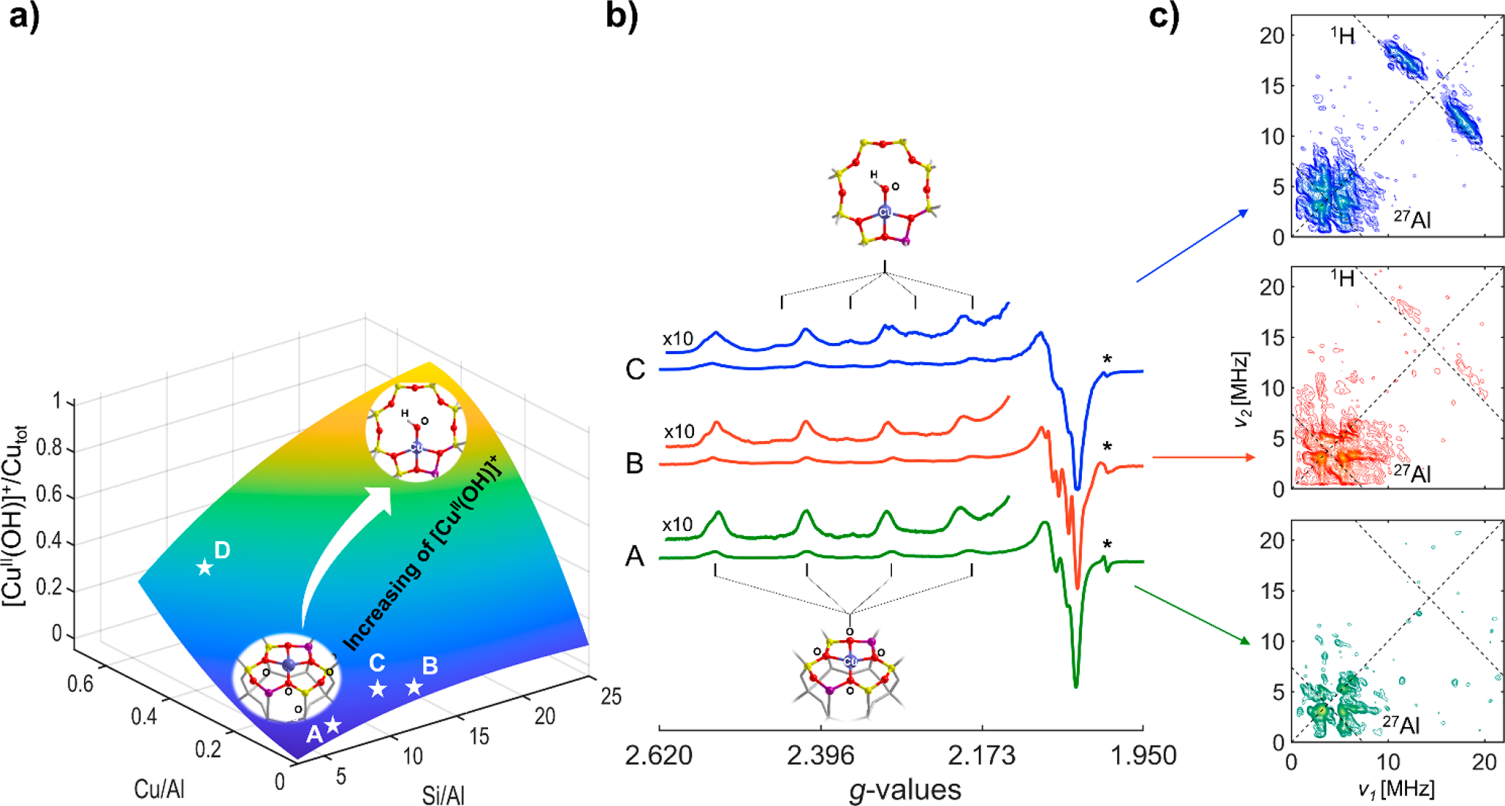
(a) Computed Cu speciation as a function of zeolite chemical composition space (Si/Al and Cu/Al ratio). The diagram is drawn based on data taken from ref (15). The compositions of the synthesized Cu-CHA samples are indicated by white stars, labeled A–D, corresponding to: A: Si/Al = 7, Cu/Al = 0.001; B: Si/Al = 15, Cu/Al = 0.005; C: Si/Al = 12, Cu/Al = 0.09; D: Si/Al = 12, Cu/Al = 0.67. (b) CW-EPR spectra of O_2_-activated CuCHA samples measured at 77 K. The asterisk indicates a carbon radical signal. Stick diagrams indicate the spectral features associated with [Cu^II^(O-6MR)_4_] in A and [Cu^II^(OH)]^+^ in B and C. The spectrum of the sample with composition Si/Al = 12, Cu/Al = 0.67 (label D in Figure 1a) is shown in Figure S1. (c) X-band ^1^H HYSCORE spectra recorded at a magnetic field position corresponding to *g* = 2.072 (*g*_⊥_) and *T* = 10 K. Experimental details are given in the Supporting Information.

The CW-EPR spectrum of Cu-CHA with composition Si/Al = 7; Cu/Al = 0.001 (spectrum A in Figure 1b) shows the characteristic hyperfine structure due to coupling of the electronic spin *S* = 1/2 of the Cu^II^ ion with the nuclear spin *I* = 3/2 of both copper isotopes ^63^Cu and ^65^Cu (natural abundances 69.17% and 30.83%, respectively) with *g*_||_ > *g*_⊥_ > *g*_e_. Computer simulation of the spectrum (Figure S1, Table 1 and Table S1) reveals the contribution of a single species with spin-Hamiltonian parameters corresponding to the [Cu^II^(O-6MR)_4_]. Previous work based on ^17^O EPR assessed the singly occupied molecular orbital (SOMO) character to be predominantly Cu d_*x*^2^*–y*^2^_ with a covalent contribution of about 30% from the coordinating framework oxygen donor atoms.^25^ Quantitative assessment indicates that the EPR-active Cu is 84% of the total Cu content. On increasing the Cu loading, the amount of EPR-active Cu^II^ decreases due to the formation of antiferromagnetic dimer species or Cu reduction (Section S3 Supporting Information). The EPR spectrum of sample B (Figure 1b) shows the presence of at least two Cu^II^ species with similar parameters (Table 1 and Table S1) amenable to [Cu^II^(O-6MR)_4_] with slightly different local environment^21,25^ and accounting for 78% of total Cu. Moving toward a composition favoring the presence of [Cu^II^(OH)]^+^ species (point C in Figure 1a), a new quartet of lines appears in the low-field region of the spectrum, highlighted by the stick diagram shown in the top of Figure 1b. Anticipating the results of this study, we assign these features to framework-bound dehydrated [Cu^II^(OH)]^+^ species. The spin-Hamiltonian parameters of the new species, assessed via computer simulations (Figure S1), are characterized by reduced *g*_||_ = 2.290 and *A*_||_ = 410 MHz values (Table 1 and Table S1), still in agreement with a dominant metal d_*x*^2^*–y*^2^_ character of the SOMO (see Section S5 Supporting Information). These values remain constant in the interval 10 K–RT, and the species corresponds to 14% of total Cu, in good agreement with data from refs (8 and 12). At higher Cu loading (sample D, Si/Al = 12 and Cu/Al = 0.67) the amount of EPR-active Cu^II^ falls to 45% of total Cu, while the contribution of [Cu^II^(OH)]^+^ is 16%. We remark that this value corresponds *only* to framework-bound isolated species that did not undergo dimerization or reduction under the adopted experimental conditions. Further details on the quantitative aspects and comparison with literature data are reported as supporting material (Section S3 Supporting Information)

**Table 1:** Experimental and Computed *g*_||_ and *A*_||_ (Absolute Values in MHz) of the Principal Cu^II^ Species in O_2_-Activated CuCHA^a^

	CHA sample	*g*_||_	*A*_||_	weight
experimental^b^	A	2.352	470	100%
	B	2.352	470	85%
		2.325	490	15%
	C	2.352	470	65%
		2.290	410	20%

	model	*g*_||_	*A*_||_	
computed^c^	[Cu^II^(O-6MR)_4_]	2.282	455	
	[Cu^II^(OH)-	2.275	388	
	(O-8MR)_3_]			

a Full tensors are reported in Table S1.

b Uncertainties of 0.004 and 5 MHz are estimated for *g*_||_ and *A*_||_.

c Computed values were obtained at the B2PLYP/CP(PPP) level of theory.

The distinctive feature of the [Cu^II^(OH)]^+^ species is expected to be the ^1^H (*I* = 1/2) hyperfine coupling of the coordinated hydroxo group. This is too small to be detected in standard CW-EPR experiments but can be measured by hyperfine techniques, which allow recovering the nuclear magnetic resonance (NMR) transitions of magnetically active nuclei coupled to the unpaired electron spin, with sub-MHz resolution.

Hyperfine sublevel correlation (HYSCORE) experiments were therefore used to interrogate each sample, and the spectra, recorded at a magnetic field corresponding to the *g*_⊥_ component of the CW-EPR spectrum, are presented in Figure 1c. We remark that under the experimental conditions Cu-coordinated water is absent^8,13^ and the only source of significant ^1^H hyperfine coupling must arise from coordinated hydroxo ligands. All HYSCORE spectra display a complex signal in the low-frequency region of the spectrum due to the interaction of the unpaired electron with a nearby ^27^Al nucleus (*I* = 5/2).^26^ While no ^1^H features are present in the HYSCORE spectrum of sample A (Si/Al = 7, Cu/Al = 0.001), a pair of elongated cross-peaks centered at the ^1^H Larmor frequency and separated by approximately 13 MHz starts to appear in sample B (Si/Al = 15, Cu/Al = 0.005) and dominates the spectrum of sample C (Si/Al = 12, Cu/Al = 0.09) in Figure 1c. Computer simulation of HYSCORE spectra recorded at different magnetic field settings allowed recovering the full ^1^H hyperfine tensor **A**_H_ = [−13.0 −4.5 +11.5] MHz (Table 2), whereby the absolute signs of the hyperfine tensors have been assigned according to *ab initio* computations (*videinfra*).

**Table 2:** ^1^H Experimental and Computed Values of Monomeric Hydroxo Cu^II^ Species in Cu-CHA^a^

^1^H	*a*_iso_	*T*_1_	*T*_2_	*T*_3_	α, β, γ
experimental^b^	–2.0	–11.0	–2.5	13.5	*, 14, 93
computed^c^	–1.1	–12.1	–1.2	13.4	–127, 26, 94

a Hyperfine constants are in MHz; Euler angles in degrees.

b Uncertainties of 1 MHz and 10° are estimated for the hyperfine couplings and Euler angles, respectively. *α angle was found immaterial.

c The computed values were obtained at the DLPNO-CCSD/cc-pwCVQZ level of theory. Euler angles relating ^H^**A**- and **g**-tensors were calculated at the B2PLYP/EPR-III level of theory.

HYSCORE experiments indicate that large proton couplings, amenable to Cu coordinated to hydroxo ligands, emerge at zeolite compositions in agreement with predictions based on the distribution reported in Figure 1a. To correlate the observed ^1^H hyperfine coupling with the specific EPR features observed in sample C, selective HYSCORE experiments have been carried out at EPR transitions pertaining exclusively to such species (Figure 2a–c). Figure 2 shows the electron spin echo (ESE)-detected EPR spectrum of sample C along with the simulation of the two main contributing species ([Cu^II^(O-6MR)_4_], green line, and [Cu^II^(OH)]^+^, in orange) identified by simulation of the continuous wave (CW) experiments (Figures S1 and S4). The spectrum corresponds to the absorption of the CW-EPR signal, and the simulated components show that the spectral features of the two species overlap at high field (*g*_⊥_), while they are sufficiently separated at low field to allow for selective excitation of the ^1^H NMR transitions. The HYSCORE spectrum recorded at position 1 (Figure 2a) shows no trace of ^1^H coupling, while clearly showing the expected ^27^Al signal. The corresponding spectrum recorded at position 2 (Figure 2b), coinciding with the *m*_I_ = −3/2 transition of the second Cu^II^ species (orange trace) shows the distinct ^1^H cross-peaks, allowing the unambiguous assignment of this species to the monomeric hydroxo-Cu^II^ species.

**Figure 2:**
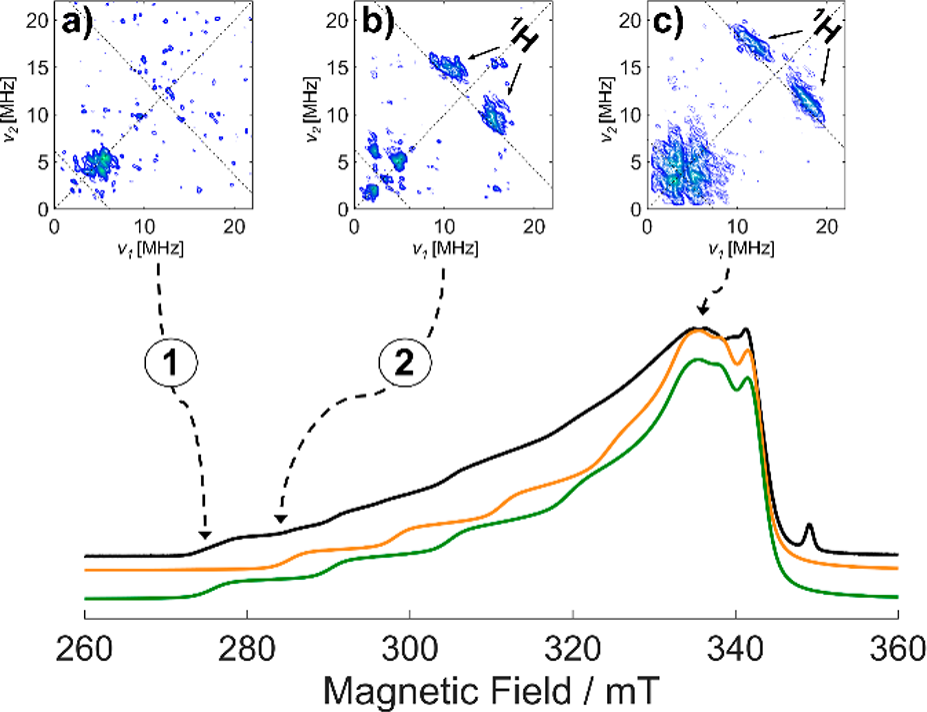
ESE-detected EPR spectrum of sample C (Si/Al = 12, Cu/Al = 0.09). Simulation of the two main species contributing the spectrum is shown in yellow ([Cu^II^(OH)]^+^) and green ([Cu^II^(O-6MR)_4_]). (a–c) HYSCORE spectra recorded at the magnetic field positions indicated by the arrows and *T* = 10 K. Experimental details are given in the Supporting Information.

To translate the spectroscopic features extracted from the analysis of the EPR and HYSCORE experiments into a microscopic structure of [Cu^II^(OH)]^+^ species in Cu-CHA, we performed *ab initio* calculations at different levels of theory on periodic and cluster models (Supporting Information Section S5). The CHA lattice is made up of cages connected through adjoining 8MR windows, which have been proposed as privileged sites for stabilizing [Cu^II^(OH)]^+^.^11,23,27^ The optimized structure of the mono-copper hydroxo species is shown in Figure 3a (see also Figure S7) and displays a 4-fold coordination, with three zeolite lattice oxygens from the 8MR (O-8MR) and the OH^–^ ligand pointing toward the center of the cage. We point out that this coordination geometry ([Cu^II^OH(O-8MRs)_3_]) is consistent with the Cu d_*x*^2^*–y*^2^_ character of the SOMO (see Figure 3b and Figure S5) and differs from previous assignments, based on trigonal Cu coordination.^13,21,22^ The computed spin Hamiltonian parameters consistently reproduce the ^1^H coupling tensor detected experimentally together with the Cu^II^**g**- and **A**-tensors (see Figure S6 and Tables 1 and 2), reflecting the decrease of the *g*_||_ and *A*_||_ components with respect to the [Cu^II^(O-6MR)_4_] species (Section S2.1 Supporting Information). The calculation of the ^1^H hyperfine tensor is particularly delicate, requiring the careful scrutiny of the different levels of theory (see Table S4). As an independent validation, the OH stretching and bending frequencies for the structure presented in Figure 3 were calculated, providing values (3658 and 928 cm^–1^) in agreement with experimental results.^9,13^ Computed values for the trigonal structure (Section S7 Supporting Information) are inconsistent with the experimental findings, providing ^1^H couplings offset by 1 order of magnitude and validating the four-coordinated model presented in Figure 3.

**Figure 3:**
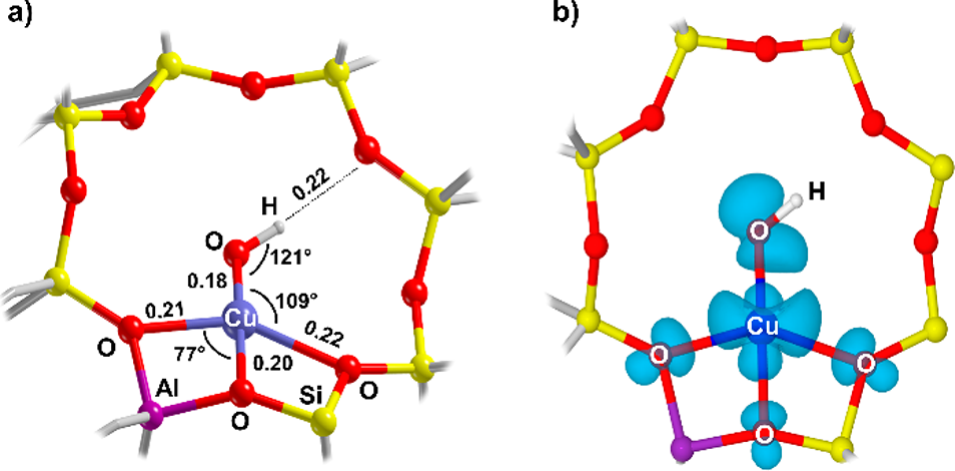
(a) Atomistic structure of [Cu^II^(OH)]^+^ species in CHA. The relevant bond distances (in nm) and angles are reported. (b) Spin density plot (isovalue of 0.002) of [Cu^II^(OH)]^+^ species in CHA.

This structure corresponds to an energy minimum, coherent with spectroscopic data collected in the temperature interval 10 K–RT. We remark that spectroscopic data collected at high temperature (673 K)^13,23^ may not be capable of distinguishing between the four and three coordination geometry due to fluctuations in Cu coordination at this temperature. In conclusion we provide a quantitative assessment of the electronic and geometric structure of monomeric hydroxo-Cu^II^ species in Cu-CHA, which can be described in terms of 4-fold-coordinated [Cu^II^(OH)(O-8MR)_3_] complexes at 8MR cages. By combining HYSCORE experiments with advanced modeling, the EPR signature of such species is univocally identified. These results highlight the potential of advanced EPR techniques in the characterization of Cu species in zeolites and provide new structural data to enable structure–function correlation in Cu-loaded zeolites.

## Supporting Information Available

Experimental procedures, computational details, simulation of EPR spectra, and details on the EPR quantification and on the computation of spin Hamiltonian parameters (PDF)
