# Supplementary material for: The Structure of Monomeric Hydroxo-CuII Species in Cu-CHA. A Quantitative Assessment
Source: J Am Chem Soc. 2022 Jul 12;144(29):13079–83. doi: 10.1021/jacs.2c06037 (PMC9335873; doi:10.1021/jacs.2c06037)
Supplement: Supplementary file 1 — ja2c06037_si_001.pdf [file ja2c06037_si_001.pdf]

The Structure of Monomeric Hydroxo−Cu^{II} Species in Cu-CHA. A Quantitative Assessment.

*Paolo Cleto Bruzzese,[†‡] Enrico Salvadori,[‡] Bartolomeo Civalleri,[‡] Stefan Jäger,[§] Martin Hartmann,[§] Andreas Pöppl,[†] and Mario Chiesa[‡]**

[†]Felix Bloch Institute for Solid State Physics, Leipzig University, 04103 Leipzig, Germany.

[‡]Department of Chemistry and NIS Centre of Excellence, University of Turin, 10125 Torino, Italy.

[§]Erlangen Center for Interface Research and Catalysis (ECRC), FAU Erlangen-Nürnberg, 91058 Erlangen, Germany.

Corresponding author: mario.chiesa@unito.it

Supporting Information

Table of Contents

S1. Materials and Methods S3

S1.1 Samples Preparation S3

S1.2 Samples Treatment S4

S1.3 EPR measurements S5

S1.4 Periodic and cluster models S5

S1.5 Computational details S6

S2. Simulations of CW-EPR spectra of O_2-activated Cu-CHA samples S8

S2.1 Correlation Plot for Copper species in dehydrated Cu-CHA S9

S3. EPR quantification of isolated Cu^{II} species in O_2-activated Cu-CHA samples S11

S3.1 EPR silent copper species S13

S4. Simulations of ^1H HYSCORE spectrum of O_2-activated Cu-CHA(C) S14

S5. Prediction of EPR parameters in $[Cu^{II}(OH)(O\text{-}8MRs)_3]$ model S15

S5.1 Comparison of spin density computed at different level of theory S15

S5.2 Computation of spin-Hamiltonian parameters in $[Cu^{II}(OH)(O\text{-}8MRs)_3]$ cluster model at different levels of theory S17

S6. Comparison of computed properties for two conformations of $[Cu^{II}(OH)(O\text{-}8MRs)_3$ model S19

S7. Comparison of computed properties for a trigonal planar $[Cu^{II}(OH)(O\text{-}8MRs)_2$ and the four-coordinated $[Cu^{II}(OH)(O\text{-}8MRs)_3$ structure. S20

S8. Simulations of ^1H HYSCORE spectrum of O_2-activated Cu-CHA(C) by using computed spin-Hamiltonian parameters S23

S9. References S24

S1. Materials and Methods

S1.1 Samples Preparation

- **Cu-CHA(A)**. Na-CHA was synthesized using the procedure reported by Fickel and Lobo.[1] 12 g of H_2O, 0.16 g of NaOH (Fisher Scientific) and 5 g of sodium silicate (Sigma Aldrich, 26.5 wt% SiO_2 , 10.6 wt% Na_2O) were mixed and stirred for 15 minutes; then 0.5 g of NH_4-Y (Zeolyst CBV100, Si/Al = 2.47) were added to the solution and stirred for 30 minutes. After that, 0.8 g of N,N,N-trimethyl-1-adamantammonium hydroxide (TMAdaOH) (25 wt%) were added to the solution and stirred for other 30 minutes. The resulting solution was transferred into Teflon-lined steel autoclave and heated at 413 K for 6 days. The product was recovered by centrifugation, washed more times with deionized water, dried overnight at 348 K and calcined in air at 823 K for 8 hours to remove the TMAdaOH. The resulting zeolite was a pure Na-CHA (Si/Al=7). The introduction of Cu^{II} ions was performed by following the procedure reported by Kevan et al.[2] The protonated form of the CHA was prepared after ion exchange with a 10 % solution of ammonia nitrate, air-drying and calcined at 773 K in air to drive-off the ammonia. Thus, 0.5 g of the zeolite were stirred with a solution composed of 5 mL of 2 mM $Cu(NO_3)_2$ and 50 mL of deionized water at about 343 K for 1 hour. The sample was recovered by centrifugation and washed with boiling water to remove Cu^{II} ions from the exterior surface. The elemental percentage composition of the final sample, determined by ICP-AES analysis, is the following: 33.35 wt% of Si, 4.76 wt% of Al and 0.01 wt% for Cu. Hence, the Cu/Al ratio is 0.001.
- **Cu-CHA(B)**. The complete synthesis of this sample was already described in Ref. 42. In brief, Na-CHA was prepared following the procedure published in the patent literature.[3] The resulting synthesis gel was transferred to a Teflon-lined steel autoclave and heated to 413 K for 6 days. The product was recovered by centrifugation, washed several times with deionized water, dried overnight at 348 K and calcined in air at 823 K for 8 h to remove the TMAdaOH. The resulting zeolite was a pure Na-CHA (Si/Al = 15) without FAU impurities. Prior to the copper exchange, the protonated form of the zeolite was obtained by liquid ion exchange with a 10% solution of ammonia nitrate, drying in the oven (348 K) overnight and heating at 823 K for 3 h to remove the ammonia residues from the framework. Cu ion-exchange was performed by following the same recipe described for Cu-CHA(A). The elemental percentage composition of the sample, determined by ICP-AES analysis, is the following: 43.70 wt% of Si, 2.72 wt% of Al and 0.03 wt% for Cu. The Cu/Al ratio is thus 0.005.
- **Cu-CHA(C)**. K/Na-CHA was synthesized through interzeolite conversion method employing NH_4-Y (Zeolyst CBV720, Si/Al = 14) as Si and Al source, TMAdaOH (25 wt%) as organic structure directing agent and NaOH and KOH as mineralizing

agents. The mixture was constructed by stirring NaOH, KOH and the TMAdaOH followed by the addition of zeolite Y. The following gel composition was adopted: SiO_2 : 1 Al_2O_3 : 0.067 TMAdaOH : 0.220 Na_2O : 0.037 K_2O : 0.012

The mixture was dried at 348 K overnight in convection oven and afterwards put in a steam sterilizer where the crystallization occurred via steam assisted crystallization procedure (T=413 K for 24 hours). The product was retrieved by centrifugation, washed four times with deionized water, dried overnight and then calcined in air at 823 K for 8 hours. The resulting zeolite was in K/Na-CHA (Si/Al=12). The ion-exchange with Cu^{II} was carried out by slightly modifying the procedure described for Cu-CHA(A). After obtaining the H- form of the zeolite (as described for Cu-CHA(A)), 1 g of the zeolite were stirred with a solution composed of 10 mL of 5mM $Cu(NO_3)_2$ and 100 mL of deionized water at about 343 K for 2 hours. The sample was retrieved by centrifugation and washed with boiling water to remove Cu^{II} ions from the exterior surface. The elemental percentage composition of the final sample, determined by ICP-AES analysis, is the following: 37.89 wt% of Si, 2.82 wt% of Al and 0.62 wt% for Cu. Hence, the Cu/Al ratio is 0.09.

Cu-CHA(D). The H-form of this zeolite comes from the same batch synthesized for Cu-CHA(C). The difference between the two samples is due to the different procedure and amount of Cu^{II} ions exchanged. For Cu-CHA(D), a modified version of the procedure reported by Giordanino et al. (Ref. 18 of the main text) was followed to introduce the Cu^{II} ions into the CHA framework. 125 mL of a solution 6mM of $Cu(CH_3COO)_2$ were mixed with 0.5 g of chabazite for 24 hours at 343 K. The exchanged zeolite was recovered by centrifugation, dried overnight at 343 K and calcined at 773 K in static air for 3 h in order to remove the residual ligands. The elemental percentage composition of the final sample, determined by ICP-AES analysis, is the following: 34.55 wt% of Si, 2.69 wt% of Al and 4.23 wt% for Cu. Hence, the Cu/Al ratio is 0.67.

S1.2 Samples Treatment

All samples were activated using the same conditions according to the following procedure. About 25 mg of Cu-CHA were put into a cell employed for the EPR measurements. Once reached an equilibrium pressure $<10^{-4}$ mbar, the cell was filled with O_2 (about 200 mbar at equilibrium pressure) in order to create a oxidative atmosphere during the dehydration step. The sample was then heated at 523 K for 2 hours (heating ramp 5 K/min). After the treatment the cell was evacuated under dynamic vacuum ($<10^{-4}$ mbar) at the same temperature (523 K) for 1 hour.

S1.3 EPR measurements

X-band (microwave frequency 9.45 GHz) CW-EPR spectra were acquired at 77 K on a Bruker EMX spectrometer equipped with an ER 4119 HS cylindrical cavity and on a Bruker EMXmicro spectrometer. In both cases, modulation frequency of 100 kHz, a modulation amplitude of 1 mT, and a microwave power of 2 mW were adopted. Pulse EPR measurements were performed at 10 K at X-band (microwave frequency 9.75 GHz) on a Bruker ELEXYS 580 spectrometer equipped with helium gas-flow cryostat from Oxford Inc.

X-band electron-spin-echo (ESE) detected EPR spectra were acquired with the pulse sequence *π/2-τ-π-τ-echo*. The pulse lengths of $t_{\pi/2}$ = 16 ns, t_{π} = 32 ns and a τ value of 200 ns were used in conjunction of a shot repetition time of 3.55 KHz.

X-band Hyperfine Sublevel Correlation (HYSCORE)[4] spectroscopy measurements were carried out with the standard pulse sequence $\pi/2$-τ-$\pi/2$-t_1-π-t_2- $\pi/2$- τ-*echo*, employing a eight-step phase cycle for deleting unwanted echoes. Pulse lengths $t_{\pi/2}$ = 16 ns, t_{π} = 32 ns and a shot repetition time of 1.77 KHz were used. The increment of the time intervals t_1 and t_1 was 16 ns, starting from 80 to 2704 ns giving a data matrix of 170x170. The τ value used for each measurements are reported in figure captions. The time traces of HYSCORE spectra were baseline corrected with a third-order polynomial, apodized with a hamming window and zero-filled to 2048 points. After 2D Fourier transformation, the absolute-value spectra were calculated.

All the EPR spectra were simulated by using the Easyspin package (version 6.0.0 dev 34)[5] running in Matlab.

S1.4 Periodic and cluster models

Cu-CHA periodic model was built starting from purely siliceous chabazite structure composed by a rhombohedral lattice with 12 tetrahedral (T) sites per unit cell. Subsequently, one Si atom of the 8MR window was substituted by one Al atom causing the removal of all the symmetry of the system (space group *P1*). The resulting model accounts for a Si/Al ratio of 11. The excess of negative charge was exactly compensated by introducing one Cu^{II} cation and a OH^- group inside the unit cell. Supercell models (2x2x1) were also employed to account for the dilution of the Cu centers in the considered samples. For these cases, a Cu/Al ratio of 0.5 was achieved by inserting 2 Al sites and 1 $[Cu^{II}OH]^+$ species per unit cell. In this way, 1 Al site was charge compensated by an acidic proton (Brønsted site) whereas the second one was compensated by the copper hydroxyl species. A full optimization (both atomic coordinates and lattice parameters) was performed.

Molecular cluster calculations were carried out to compute **g**-tensor, hyperfine interactions (*hfi*) of Cu^{II} species and ^1H nucleus as well as the orientation of the ^1H

hyperfine tensor with respect to the **g** frame. Cluster models were cut out from the related optimized periodic structure and the dangling bonds were saturated with hydrogen atoms oriented along the broken bonds. No further geometry optimization was performed in order to maintain the same relaxed atomic coordinates as in the optimized periodic structure. The net charge on the molecular models was set to 0 in a doublet spin state.

S1.5 Computational details

Periodic calculations (geometry optimization and vibrational frequencies calculations) were performed by using the distributed parallel version of CRYSTAL17 code (PCRYSTAL)[6] within the framework of Density Functional Theory (DFT) exploiting the hybrid B3LYP method, Becke's three parameters exchange functional and the correlation functional from Lee, Yang and Parr.[7,8] Dispersive interactions, extremely relevant in determining the geometry of zeolites,[9,10] were taken into account empirically through the so-called DFT-D3 method in conjunction with a three-body correction.[11,12] Pob-TZVP basis set[13] was adopted for all the elements, except for the extra-lattice O and H atoms of the hydroxyl group for which the Ahlrichs VTZP basis set[14] was employed. A pruned grid consisting of 75 radial points and a maximum number of 974 angular points in regions relevant for chemical bonding has been adopted. The accuracy of the calculation of the two electron integrals in the Coulomb and exchange series was controlled by setting truncation criteria at the values of 10^{-7} except for the pseudo-overlap of the HF exchange series which was fixed to 10^{-25}. A shrink factor equals to 6 was used to diagonalize the Hamiltonian matrix in at least 112 k-points of the first Brillouin zone. The default value of mixing (30%) of the Kohn-Sham (KS) matrix at a cycle with the previous one was adopted. The threshold in energy variation of SCF cycles was set equal to 10^{-8} Hartree for geometry optimization and equal to 10^{-10} Hartree for frequency calculations. The number of unpaired electrons in the unit cell was not locked to one in order to leave the SCF procedure to freely converge to a doublet spin state of the system wavefunction.

Harmonic vibrational frequencies were computed at the center of the first Brillouin zone in the reciprocal space (Γ point) from the diagonalization of the mass-weighted Hessian matrix of the second energy derivatives with respect to atomic displacement.[15–17] Two displacements for each atom along each cartesian direction were considered to numerically compute the second energy derivatives (NUMDERIV 2).

Anharmonic calculation of O-H bond stretching was performed by considering the selected O-H bond as a independent oscillator.[18,19] The O-H distance was varied around the equilibrium value d_0 [d_0+ (-0.2, -0.16, -0.06, 0.00, 0.16, 0.24, 0.3 Å)]. All the other geometrical features were kept fixed. For each value of the O-H distance the total potential energy was computed and a polynomial curve of sixth degree was used to best fit the energy points (root mean square error below 10^{-6} Hartree). Thus, the corresponding mono-dimensional nuclear Schrödinger equation is solved numerically[20] according to the procedure proposed by P. Ugliengo.

Molecular cluster calculations (computation of **g**- and **A**-tensors and their relative orientations) were carried out with ORCA (v4.2.1 and v5.0.2) code.[21,22] The spin-orbit coupling (SOC) contribution (not negligible for Cu^{II} species)[23] was explicitly treated by using complete mean-field spin-orbit operator (SOMF).[24] The potential was constructed to include one-electron terms, compute the Coulomb term in a semi-numeric way, incorporate exchange via one-center exact integrals including the spin-other orbit interaction and include local DFT correlation (SOCFlags 1,2,3,1 in ORCA). The specialized CP(PPP) basis set[25] was employed for Cu nucleus for all the calculations, while the def2-TZVP basis sets[26] were employed for Si, Al, O and H atoms. Concerning DFT calculations (hybrid and double hybrid), the H atom from the OH^- group was treated with the well-known EPR-III basis set.[27] Increased integration grids were employed (DefGrid3 in ORCA v5.0.2 and Grid7 in ORCA v4.2.1 nomenclature) and tight energy convergence settings were applied throughout (TightSCF). The resolution of identity (RI) in conjunction with the corresponding auxiliary basis sets were adopted. In case no auxiliary basis set was available, the AutoAux keyword was employed to automatically build the auxiliary basis set. Double-hybrid calculations were carried out by using the "relaxed" Møller-Plesset (MP2) density and keeping all the electron active (NoFrozenCore keyword).

Open-shell domain-based local pair natural orbital coupled cluster with single and double excitations (DLPNO-CCSD) calculations[28] were performed to compute the ^1H hfi of the hydroxo group bound to Cu^{II}. The CCSD calculations were initiated with unrestricted Kohn-Sham orbitals and followed the standard DLPNO procedure.[29,30] The specific settings employed for this method are the followings: TCutMKN=1.0×10^{-4}, TCutDO=5.0×10^{-3}, TCutPNO=1.0×10^{-7}, TCutPNOSingles=0.00, TScalePNOCore=1.0×10^{-3}, and TScalePNOSOMO=1.0×10^{-1}. All the electrons were set as active and "unrelaxed" spin density was adopted (where the effect of orbital relaxation is recovered only through the action of $\exp(T_1)$ onto the reference).[28] Concerning CCSD calculations, all the elements (apart from Cu) were treated with cc-pwCVQZ basis sets[31,32] and its corresponding auxiliary basis sets. Scalar relativistic calculations were also performed with the second-order Douglas-Kroll-Hess (DKH2)[33–35] and the DKH-recontracted basis. The property operator was relativistically modified in the picture-change framework[36–38] and the nuclei were treated as Gaussian distributions in the finite nucleus model. However, since the computed hyperfine couplings of ^1H with scalar relativistic approach does not significantly differ from the nonrelativistic ones, they will not be commented here. The maximum amount of memory per core used for these calculations was 62.5 GB.

S2. Simulations of CW-EPR spectra of O_2-activated Cu-CHA samples

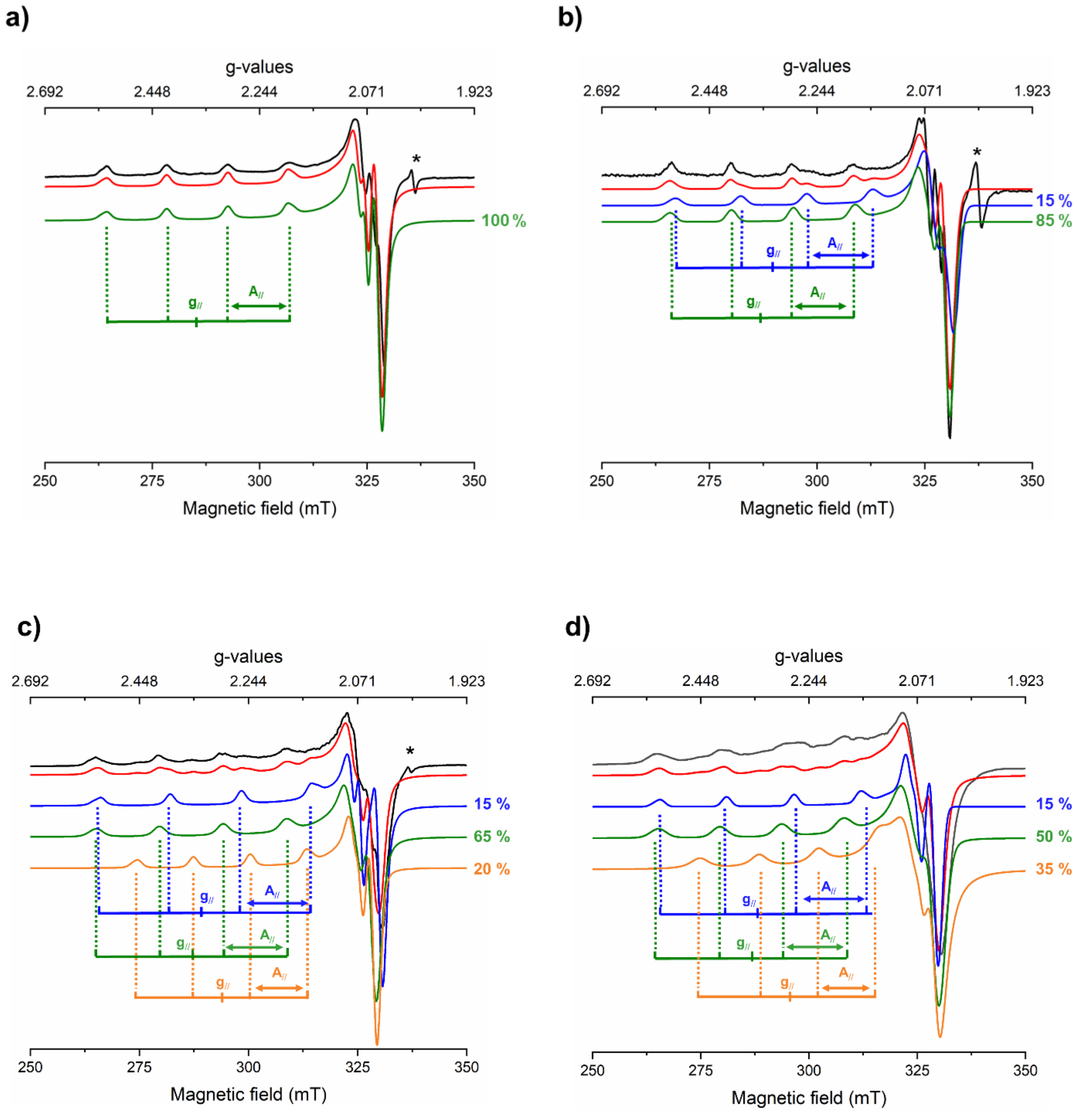

Figure S1. Experimental (black lines) and computer simulations (red lines) of CW-EPR spectra recorded at 77 K of O_2-activated a) Cu-CHA(A) (Si/Al=7; Cu/Al=0.001), b) Cu-CHA(B) (Si/Al=15; Cu/Al=0.005), c) Cu-CHA(C) (Si/Al=12; ; Cu/Al=0.09), d) Cu-CHA(D) (Si/Al=12; ; Cu/Al=0.67). The contribution of each individual Cu species together with their weight used for the overall simulations are shown in green, blue and orange for $[Cu^{II}(O\text{-}6MR)4]_a$, $[Cu^{II}(O\text{-}6MR)4]_b$ and $[Cu^{II}(OH)(O\text{-}8MR)_3]$.

Samples	Si/Al; Cu/Al	Weight	Species	$g_\perp$	$g_{//}$	$A_\perp$	$A_{//}$	lwpp Gaussian	lwpp Lorentzian
Cu-CHA(A)	7; 0.001	100 %	$[Cu^{II}(O\text{-}6MR)_4]_a$	2.075	2.352	35	470	1.8 ± 0.5	1 ± 0.2
Cu-CHA(B)	15; 0.005	85 %	$[Cu^{II}(O\text{-}6MR)_4]_a$	2.075	2.352	35	470	1.8 ± 0.5	1 ± 0.2
		15 %	$[Cu^{II}(O\text{-}6MR)_4]_b$	2.072	2.325	35	490	1.0 ± 0.3	1 ± 0.2
Cu-CHA(C)	12; 0.09	65 %	$[Cu^{II}(O\text{-}6MR)_4]_a$	2.075	2.352	35	470	1.8 ± 0.5	1 ± 0.2
		15 %	$[Cu^{II}(O\text{-}6MR)_4]_b$	2.072	2.325	35	490	1.0 ± 0.3	1 ± 0.2
		20 %	$[Cu^{II}(OH)(O\text{-}8MR)_3]$	2.072	2.290	30	410	1.5 ± 0.4	0.5 ± 0.2
Cu-CHA(D)	12; 0.67	50 %	$[Cu^{II}(O\text{-}6MR)_4]_a$	2.075	2.352	47	460	3.0 ± 0.5	0.5 ± 0.3
		15 %	$[Cu^{II}(O\text{-}6MR)_4]_b$	2.075	2.329	35	500	2.0 ± 0.3	0.0 ± 0.3
		35 %	$[Cu^{II}(OH)(O\text{-}8MR)_3]$	2.072	2.290	45	410	0.0 ± 0.3	2.7 ± 0.4

Table S1. Experimental **g**- and Cu **A**-tensors retrieved from the simulations of the CW-EPR spectra recorded at 77 K reported in Figure 1 and Figure S1. Only the absolute values of the hyperfine components are extracted from the spectra. Hyperfine couplings are given in units of MHz. Uncertainty of 0.004, 5 MHz and 6 % were estimated for the g-values, hyperfine couplings and weights, respectively. Linewidth for isotropic broadening taken peak-to-peak (lwpp) is given in mT. Peak-to-peak refers to the horizontal distance between the maximum and the minimum of a first-derivative lineshape. Species $[Cu^{II}(O\text{-}6MR)_4]_a$ and $[Cu^{II}(O\text{-}6MR)_4]_b$ were assigned according to Ref. 42 whereas the assignment of the spin-Hamiltonian parameters of species $[Cu^{II}(OH)(O\text{-}8MR)_3]$ comes from this work.

S2.1 Correlation Plot for Copper species in dehydrated Cu-CHA

As pointed out by Larsen and co-workers[39,40] the EPR spectral parameters for copper-exchanged zeolites can be discussed in the context of an empirical model developed by Peisach and Blumberg for interpreting the EPR spectra of Cu^{II} in model compounds and proteins.[41] Empirical correlations between $A_{||}$ and $g_{||}$ have been established for a series of Cu model compounds in solution with varying ligands and well-defined structures. This has enabled Cu EPR parameters to be correlated to the nature of the copper ligands and the overall charge of the complex. Such correlations have been used by Larsen et al.[39] to infer the formal charge of Cu species in zeolites. The Peisach-Blumberg correlation plot for the $[Cu^{II}(O\text{-}6MR)_4]$ and $[Cu^{II}(OH)(O\text{-}8MR)_3]$ species and the two model compounds $[Cu(H_2O)_6]^{2+}$ and $[Cu(OH)_4]^{2-}$ is shown in Figure S2.

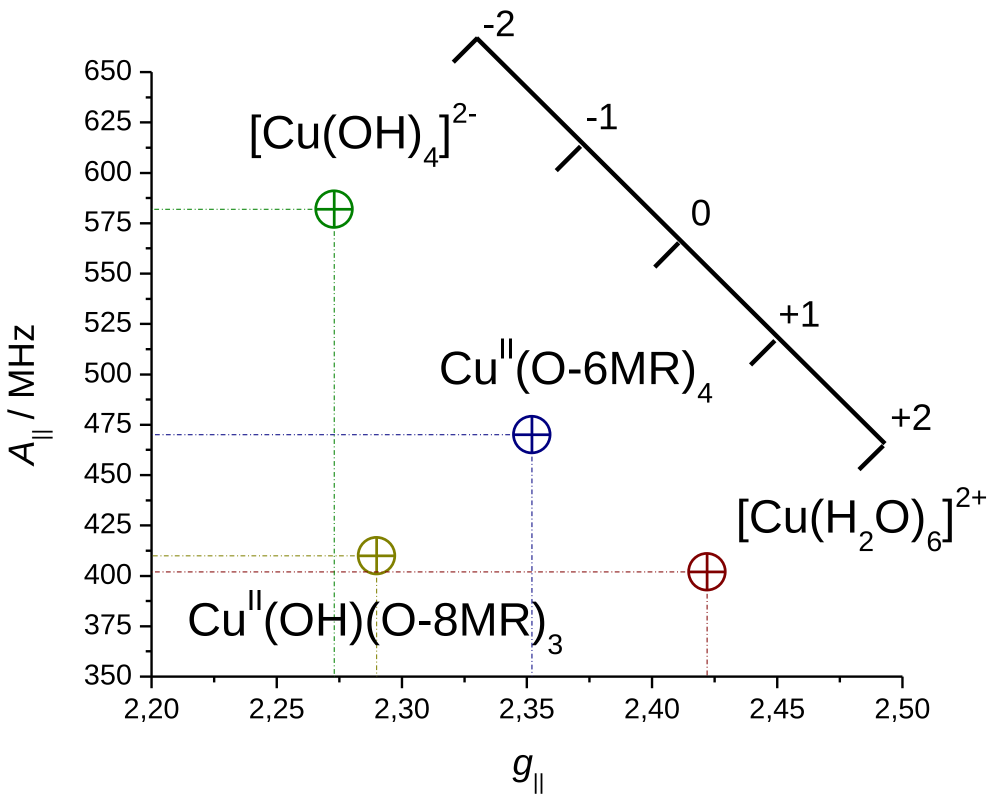

Figure S2. Peisach-Blumberg plot correlating $A_{||}$ and $g_{||}$ for the copper interfacial complexes ($[Cu^{II}(O\text{-}6MR)_4]$) and $[Cu^{II}OH(O\text{-}8MRs)_3]$ and two solution model compounds.

The parameters of the Cu^{II} complex at the 6MR position ($[Cu^{II}(O\text{-}6MR)_4]$) fall in the region where neutral oxygen coordinated copper complexes are expected. This is coherent with the computed Mulliken charge on Cu equal to +0.7 and the fact that Cu^{II} is coordinated by four O^{2-} ions and stabilized by 2 Al^{3+} ions leading to a neutral complex. We remark that the observation of a non-negligible spin density on the Al^{3+} (observed in the HYSCORE spectra reported in Figure 1 of the main manuscript and discussed in previous works by us[42]) substantiate this notion, pointing to a situation similar to the case of contact ion pairs in solutions. In a similar way, the $[Cu^{II}(OH)(O\text{-}8MR)_3]$, whereby the single positive charge is balanced by a single Al^{3+} ion, remains in this "neutral region" but shows decreased *g* and *A* values. The *g* factor is consistent with the hydroxo complex while the reduced $A_{||}$ coupling concurs with departure from a square planar complex as reported by Peisach[41] and coherent with the structure derived in this work. Importantly, the decrease of $A_{||}$ and $g_{||}$ from the framework coordinated ($[Cu^{II}(O\text{-}6MR)_4]$) to the hydroxo complex $[Cu^{II}OH(O\text{-}8MRs)_3]$ is reproduced by the theoretical calculations (Table 1 of the main manuscript).

S3. EPR quantification of isolated Cu^{II} species in O_2-activated Cu-CHA samples

Figure S3 reports a comparison between the total Cu content in the four samples studied in this work determined by ICP-AES analysis and the two paramagnetic Cu^{II} species identified in this work namely $[Cu^{II}(O\text{-}6MR)_4]_{a,b}$ and $[Cu^{II}(OH)(O\text{-}8MR)_3]$. The total amount of paramagnetic Cu^{II} species was determined by using the SpinCount package of Bruker Xenon Software.[43] Based on the difference between the amount of paramagnetic Cu^{II} measured by CW-EPR and the amount of Cu determined by ICP-AES, the percentage of EPR silent Cu is also recovered. Inspection of Figure S2 shows that the amount of paramagnetic Cu^{II} decreases as a function of increasing Cu content and Si/Al ratio. While in sample A (Si/Al=7; Cu/Al=0.001) 84% of total Cu is EPR active after dehydration, in sample C (Si/Al=12, Cu/Al=0.09) this fraction lowers to 68%. This EPR active Cu^{II} is composed by 54% of Cu^{II} at 2 Al sites and 14% of $[Cu^{II}(OH)(O\text{-}8MR)_3]$ at 1Al sites. These correspond to the isolated $[Cu^{II}(OH)]$ species that survived in their oxidized state and did not undergo condensation to form antiferromagnetically coupled dimers (see section S3.1). This amount is consistent with literature data where the amount of $[Cu^{II}(OH)]$ species was determined by XANES spectroscopy[44] or by chemical titration[45] and with the theoretical value expected from the compositional phase diagram reported by Paulucci.[46]

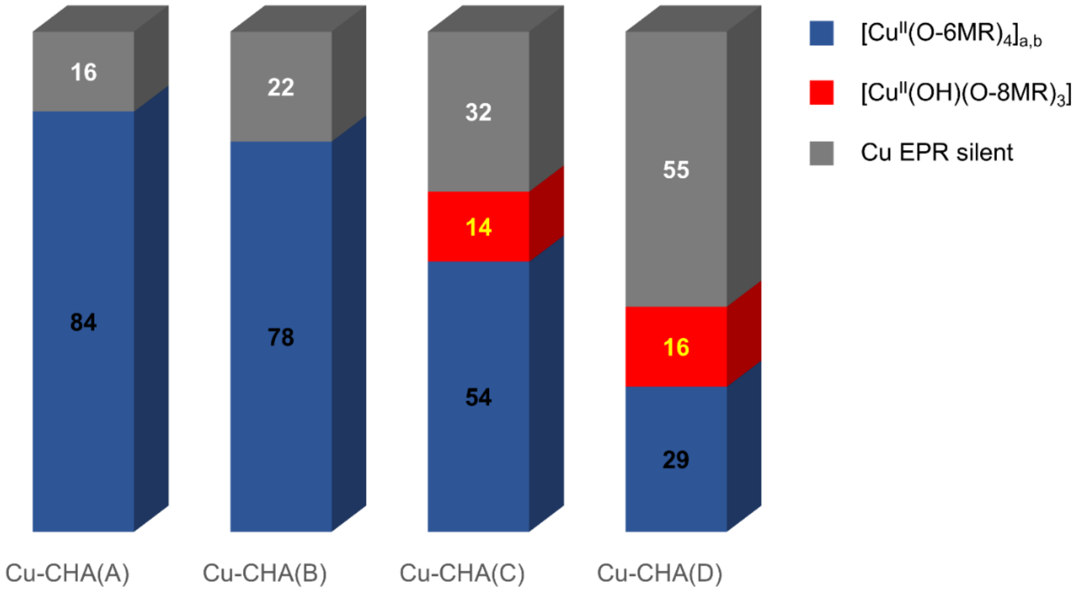

Figure S3. Column graph representing the percentage of Cu species quantified by CW-EPR with respect the total amount of Cu determined by ICP-AES. The percentage of each species are reported inside the column according to the color code given by the legend.

Finally, sample D (Si/Al=12, Cu/Al=0.67) displays the largest amount of EPR silent species after the dehydration treatment (55%), while the number of framework bound $[Cu^{II}(OH)(O\text{-}8MR)_3]$ at 1Al sites increases up to 16%. These values concur with estimates

obtained by XANES spectroscopy on a Cu-CHA sample with Si/Al=15 and Cu/Al=0.5 activated under He for which [Cu^{II}(OH)] was found to be 13% and Cu^{I} = 79%.[44] Clearly in this case reduction or condensation of [Cu^{II}(OH)] species during the outgassing treatment (1 h under high vacuum at 773 K) and the formation of EPR silent species is dominant. Assuming that the EPR silent species are originated by [Cu^{II}(OH)] their overall amount (71%) is in the range predicted by the compositional phase diagram of Paulucci and consistent with estimates present in the literature (Gao *et al*.[47,48]) and reported in Table S2.

Table S2. Speciation of Cu sites and estimation of their amount in activated Cu-CHA samples as reported in literature.

Composition	Activation procedure	Characterization technique	2Al Cu^{II}	1Al Cu^{II}OH	1Al Cu^{I}	Cu_xO_y	Reference
Si/Al = 15 Cu/Al = 0.50	773 K in He	XANES spectroscopy	8.0 %	13.0 %	79.0 %	/	Ref. 44
Si/Al = 12.5 Cu/Al = 0.29	823 K in O_2/ He flow for 1 h	H_2-TPR	45.0 %	55.0 %	/	/	Ref. 49
Si/Al = 12 Cu loading = 2.1 wt %	523 K in He for 1 h	EPR (based on the intensities difference of the hydrated and dehydrated form)	37.0 %	63.0 %	/	/	Ref. 47
Si/Al = 14 Cu/Al = unknown	523 K in O_2/ He flow for 2 h	EPR (based on the intensities differences of the hydrated and dehydrated form and the weights used for simulating the spectra)	25.0 %	50.0 %	/	25.0 %	Ref. 50
Si/Al = 14 Cu/Al = unknown	523 K in He flow for 14 h		25.0 %	/	50.0 %	25.0 %	
Si/Al = 15 Cu/Al = 0.10	773 K in O_2	Titration of the residual H^+	90.0 %	10.0 %	/	/	Ref. 45
Si/Al = 12.5 Cu/Al = 0.11	873 K in O_2 and H_2O for 5h	NO_2 adsorption + TPD and H_2-TPR	45.0 %	55.0 %	/	/	Ref. 51
Si/Al = 12 Cu/Al = 0.09	523 K in O_2 for 2 h	EPR (direct measurement)	54.4 %	13.6 %	/	/	**This work**
Si/Al = 12 Cu/Al = 0.67			29.2 %	15.7 %	/	/	

S3.1 EPR silent copper species

The loss of Cu^{II} EPR signal in Cu zeolites prepared by solution ion exchange as a function of dehydration is a well-known fact extensively reported in literature. The mechanisms leading to such a signal loss and the role played by topology, Si/Al ratio, and copper loading have been recently put in perspective by Van Bokhoven and co-workers[52] and can be summarized as follows:

1) One of the most common but poorly understood process is the so-called “autoreduction” or “self-reduction”, which takes place during the activation of copper-exchanged zeolites. It implies the transformation of some of the Cu^{II} species to Cu^{I} at elevated temperature, typically above 673 K, in an inert environment and in the absence of a reducing agent. The generally accepted pathway involves the elimination of water from two neighboring $[Cu^{II}(OH)]^{+}$ species leading to $[Cu{-}O{-}Cu]^{2+}$ species. These species feature two antiferromagnetically coupled copper ions and are EPR silent. At high temperature (>673 K) these copper-oxo bridged species decompose to Cu^{I} and molecular oxygen, which desorbs from the material. The EPR signal loss in general (and in our case in particular) can therefore be associated to the first step of the mechanism (i.e. the $[Cu^{II}(OH)]$ condensation).
2) A second possible reason for the loss of Cu^{II} EPR intensity is copper reduction by reaction with residual carbonaceous impurities, which can remain in the catalyst after synthesis. In this case conversion of EPR active Cu^{II} to EPR silent Cu^{I} occurs quantitatively at low temperature during the thermal activation with release of CO_2.[53]
3) Cu^{II} reduction involving the formation of $Cu\text{-}O^{\bullet -}$ radical species has been suggested in literature, but never experimentally confirmed.[54]
4) A final reason for the loss of Cu^{II} EPR active species that has been proposed in literature[50] is linked to the formation of three-coordinated monomeric Cu species characterized by very fast relaxation rates as a consequence of a pseudo Jahn-Teller effect. This effect has never been directly observed and the detection of $[Cu(OH)]^{+}$ with four-coordination geometry reported in this work, rules out this hypothesis, leaving the first three (in particular the first two) mechanisms as the origin of loss of EPR signal upon dehydration of Cu exchanged zeolites.

S4. Simulations of ^{1}H HYSCORE spectrum of O_2-activated Cu-CHA(C)

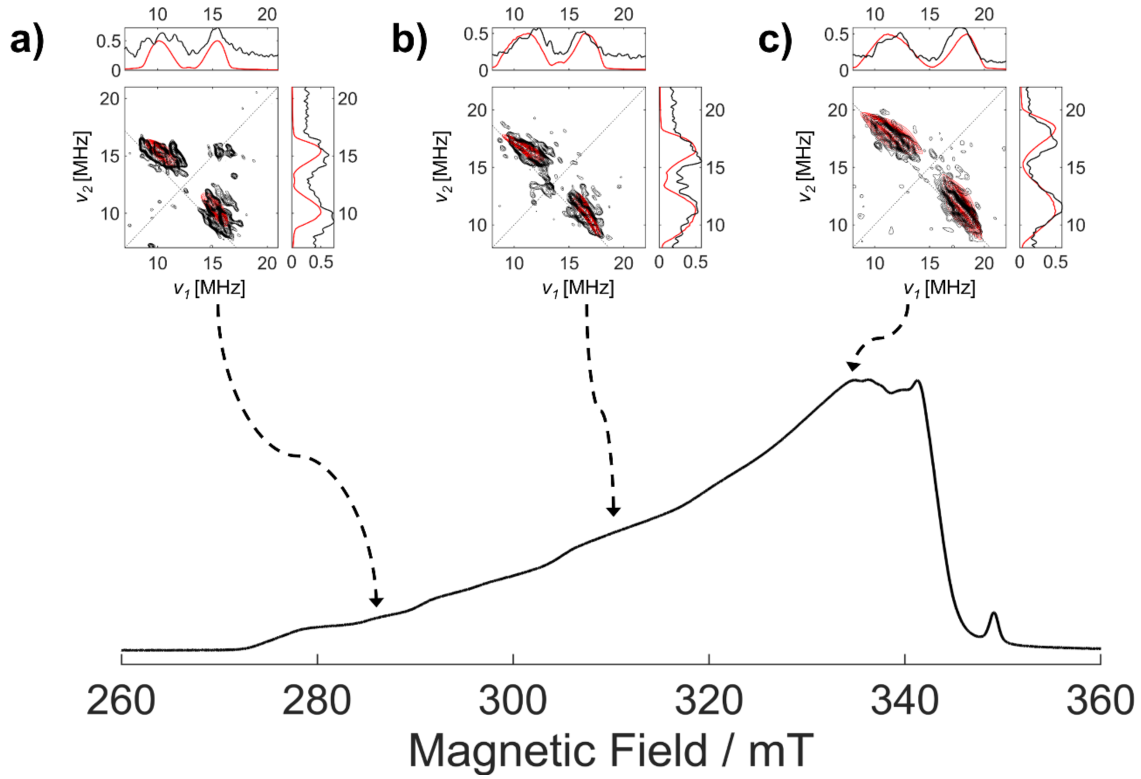

Figure S4. Experimental (in black) and computer simulations (in red) of X-band ^{1}H HYSCORE spectra of O_2-activated Cu-CHA(C) acquired at a) 284.3 mT, b) 313.0 mT and c) 333.4 mT. The corresponding sum projections are also shown. Each HYSCORE spectrum was measured by employing a τ value that suppress the signal at the proton Larmor frequency at the corresponding magnetic field (τ= 164 ns in a), τ= 150 ns in b) and τ= 130 ns in c)). The computer simulations were obtained by using the spin-Hamiltonian parameters given in Table 1 (CHA sample C) and Table 2 (simulated values) of the main text. The experimental ESE spectrum is reported in black.

S5. Prediction of EPR parameters in [Cu^{II}(OH)(O-8MRs)$_3$] model

To validate the size consistency and goodness of the cluster models extracted from the optimized periodic structure according to the procedure reported in section S1.4, the ^{1}H hyperfine couplings for a cluster and periodic [Cu^{II}(OH)(O-8MRs)$_3$] model were computed at the same level of theory.

[Cu^{II}(OH)(O-8MRs)$_3$] Model	Level of theory	^{1}H **A**-tensor			
		a_{iso}	T_1	T_2	T_3
Periodic	PBE0 (40% of HF exchange)	-1.4	-12.3	-1.9	14.2
Cluster	PBE0 (40% of HF exchange)	-1.1	-12.5	-1.4	13.9

Table S3. Comparison of ^{1}H hyperfine couplings computed for a periodic and cluster [Cu^{II}(OH)(O-8MRs)$_3$] model at the same level of theory.

The nice agreement between the two approaches validates the cluster model adopted in this work. The small differences observed between the cluster and periodic computed values are more likely attributed to the different grid schemes adopted for numerical integration by CRYSTAL and Orca codes.

S5.1 Comparison of spin density computed at different level of theory

The computation of accurate electron densities is the most important prerequisite for the quantitative reproduction of molecular properties and, thus, spectroscopic experiments. Indeed, the hyperfine structure in EPR is a direct reflection of the electron spin density distribution over the paramagnetic center and its local environment. Therefore, satisfactory results in predicting hyperfine couplings are inevitably dependent on the calculation of precise first principles spin densities.[55]

Although common hybrid DFT methods provide good results for organic radicals,[56] they usually have difficulties in treating open-shell transition metal complexes.[57,58] The failures in describing the properties of paramagnetic transition metal complexes are rooted in the use of approximate exchange-correlation functionals. They typically suffer from the so-called self-interacting error (SIE)[59] which generates a too covalent description of the metal-ligand bond. This brings to a large delocalization of the spin density, and there is less spin density on the metal atom than it should be. The inclusion of more Hartree-Fock (HF) exchange into hybrid density functional can in part compensate such tendency. Nevertheless, it does not solve the problem since the optimum mixing strongly depends on the system and, in certain cases, it can produce high spin contamination.[58]

For accurate spin densities, in cases for which a DFT description fails, *ab initio* electron correlation needs to be included. For instance, the introduction of second order perturbation theory (PT2) correlation in the double-hybrid density functionals (DHDFs)[60] has proved to provide excellent results for computing EPR parameters.[61–63] On the other hand, wave function based methods have nowadays become available also for systems of “realistic” size due to efficient algorithmic approximation. In particular, the orbital-optimized second-order Møller-Plesset perturbation theory (OO-MP2) and the DLPNO-CCSD approaches demonstrated to possess more systematic performance than DFT methods.[28,58,64]

Motivated by these concerns, we computed and plotted the spin density on a cluster model of [Cu^{II}(OH)(O-8MRs)$_3$] extracted from the optimized periodic model at three indicative levels of theory: B3LYP (Figure S5a), DSD-PBEP86[65] (Figure S5b) and DLPNO-CCSD (Figure S5c). The spin density is mainly localized in the Cu *3*$d_{x^2-y^2}$ orbital and the *2*p orbitals of the oxygen donor atoms. The oxygen donor atom of the hydroxyl group allocates more spin density than the others from the zeolite framework due to the more pronounced covalency of the Cu-OH bonding with respect to the Cu-O bonds.

However, while the amount of spin density on the oxygen donor atoms of the CHA zeolite does not differ significantly by increasing the level of theory (from B3LYP up to DLPNO-CCSD), there is a huge difference between the computed spin density over the OH group. B3LYP predicts about the 15 % of spin density on the O atom of the hydroxyl group, whereas DSD-PBEP86 and DLPNO-CCSD give very similar value of 7.1 % and 7.8 %, respectively. These findings reveal that hybrid B3LYP functional exaggerates spin delocalization towards the OH group predicting large spin polarization region between the Cu atom and the hydroxyl group and overestimating the covalency of the Cu-OH bond. On the contrary, DSD-PBEP86 and DLPNO-CCSD methods perform similarly localizing more electron spin on the Cu atom (about the 81 %) with respect to B3LYP.

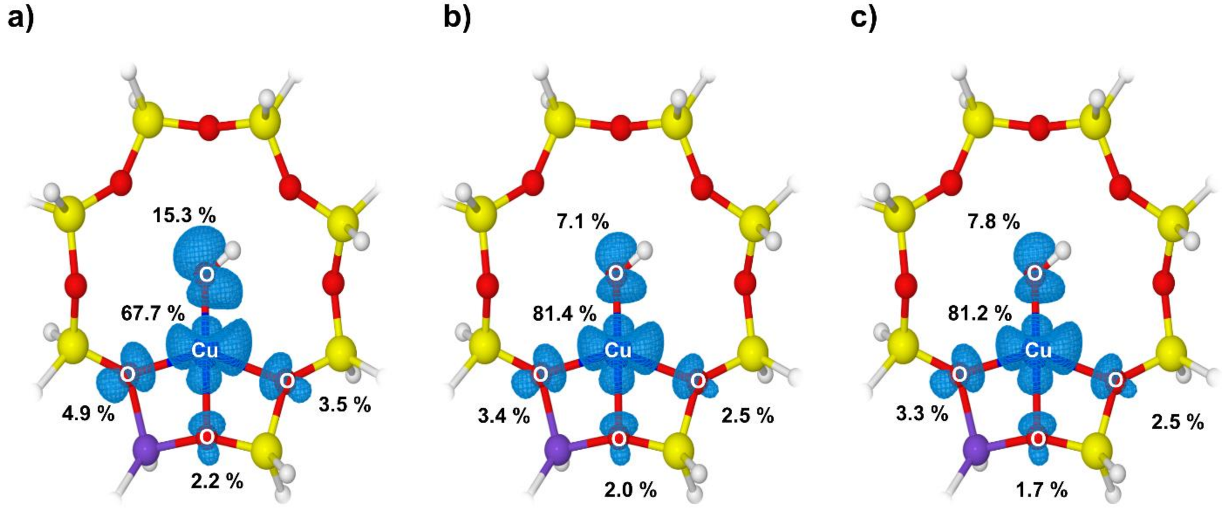

Figure S5. Spin density plots of [$Cu^{II}(OH)(O\text{-}8MRs)_3$] cluster model obtained at a) B3LYP, b) DSD-PBEP86 and c) DLPNO-CCSD levels of theory. Isovalue levels were fixed at 0.002. Löwdin spin populations of the relevant atoms are indicated in percentage. Si, Al and H atoms are indicated in yellow, violet and white, respectively.

S5.2 Computation of spin-Hamiltonian parameters in [$Cu^{II}(OH)(O\text{-}8MRs)_3$] cluster model at different levels of theory

The computed **g** and ^{1}H **A** tensors components relative to [$Cu^{II}(OH)(O\text{-}8MRs)_3$] cluster model are reported in Table S4. Popular hybrid DFT methods (B3LYP, PBE0 and B3PW91) tend to overestimate the hyperfine couplings of the ^{1}H and underestimate the g_z value because of the too covalent description of the Cu-OH bond. Highly parametrize functionals (M06 and M06-2X) produces the worst result with a too high g_z component (see M06-2X) and an overestimation of the ^{1}H hyperfine interaction (see M06). The DSD double-hybrid functionals tested here gave very similar results, not so far from the "gold standard" DLPNO-CCSD approach and the simulated values. Concerning the ^{1}H hfi, we found the DLPNO-CCSD method superior to all functionals tested so far.

However, increasing the HF exchange in the common PBE functional improves the results providing smaller dipolar couplings and $|a_{iso}|$ term for the ^{1}H nucleus. Simultaneously, g_z value increases due to the reduction of the SIE. We remark that the most performing hybrid DFT method was PBE with 40 % of HF exchange for this specific case.

Level of theory	**g**-tensor			^{1}H **A**-tensor				
	g_x	g_y	g_z	a_{iso}	T_1	T_2	T_3	α, β, γ
B3LYP	2.026	2.107	2.212	-8.0	-18.4	-2.1	20.5	-135, 25, 100
PBE0 (25% of HF exchange)	2.027	2.124	2.238	-5.7	-16.8	-1.5	18.4	-134, 26, 97
PBE (30% of HF exchange)	2.028	2.136	2.257	-3.9	-15.3	-1.4	16.7	-133, 26, 96
PBE (35% of HF exchange)	2.029	2.148	2.277	-2.3	-13.8	-1.3	15.1	-131, 26, 95
PBE (40% of HF exchange)	2.030	2.160	2.295	-0.9	-12.4	-1.4	13.8	-129, 25, 94
PBE (45% of HF exchange)	2.031	2.168	2.313	0.1	-11.2	-1.6	12.9	-126, 25, 94
PBE (50% of HF exchange)	2.032	2.175	2.328	0.8	-10.4	-1.8	12.2	-123, 24, 94
B3PW91	2.026	2.109	2.214	-8.0	-18.5	-1.9	20.4	-134, 25, 99
M06	2.026	2.286	2.861	-16.6	-19.3	-2.7	21.9	58, 54, -48
M06-2X	2.065	2.437	3.026	1.8	-9.8	-2.1	11.9	-59, 31, 86
B2PLYP	2.029	2.135	2.275	-3.0	-15.3	-1.6	16.9	-123, 25, 95
DSD-BLYP	2.035	2.185	2.416	0.82	-11.3	-1.6	13.0	-127, 25, 94
DSD-PBEP86	2.035	2.181	2.399	0.58	-11.3	-1.6	12.9	-127, 25, 94
DLPNO-CCSD*	/	/	/	-1.1	-12.1	-1.2	13.4	/
Simulated	2.072	2.072	2.290	-2.0	-11.0	-2.5	13.5	*, 14, 93

*Since the calculation of **g** tensor and relative orientation of the **A** tensor are not yet implemented in ORCA code at DLPNO-CCSD level of theory, they are not reported here.

Table S4. Spin-Hamiltonian parameters calculated at different level of theory on $[Cu^{II}(OH)(O\text{-}8MRs)_3]$ cluster model. The **A**-tensor components are given in MHz. Euler angles (α,β,γ) are given in degrees.

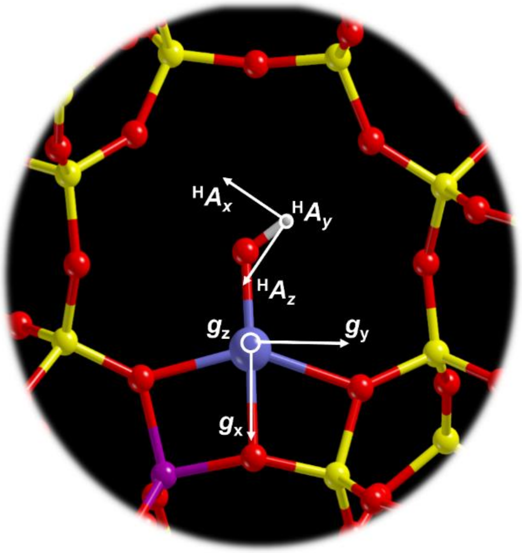

Figure S6. Computed (at B2PLYP/CP(PPP) for Cu and EPR-III for H nuclei, level of theory) **g** and ^{1}H **A** tensors orientations reported on the atomistic model of $[Cu^{II}(OH)(O\text{-}8MRs)_3]$. Cu, Si, Al, O and H atoms are shown in blue, yellow, violet and gray, respectively.

S6. Comparison of computed properties for two conformations of [Cu^{II}(OH)(O-8MRs)$_3$ model

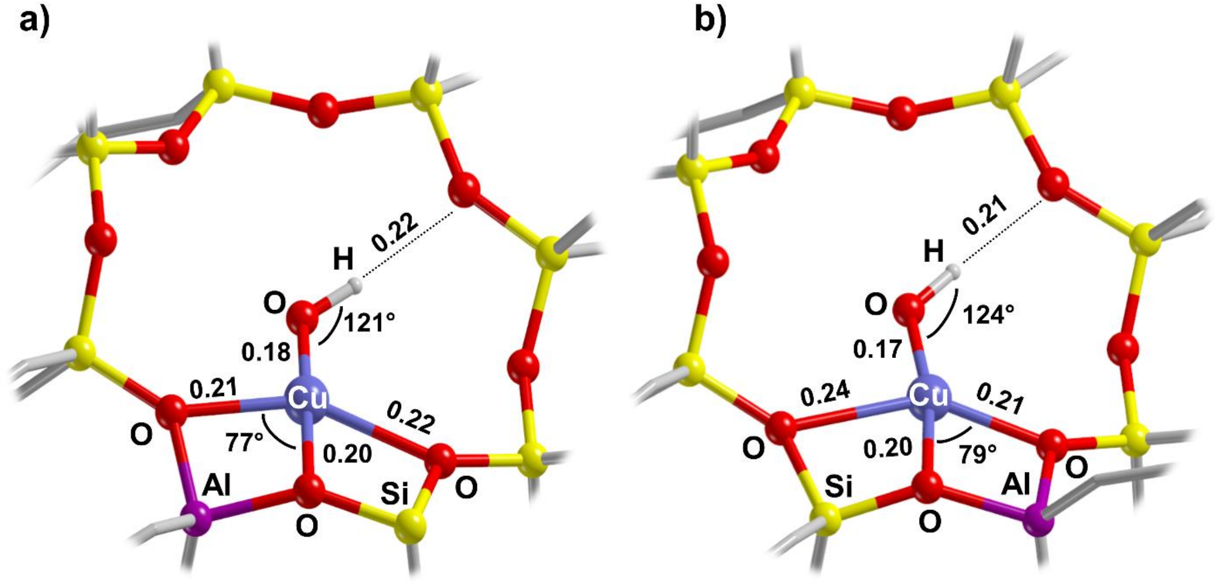

Figure S7. Atomistic structures of [Cu^{II}(OH)(O-8MRs)$_3$] model in two possible conformations. In **a)** the OH group is pointing towards the opposite side of the 8MR with respect of the Al location. In **b)** the OH group is pointing at the same side of the Al substitution in the 8MR. The relevant bond distances (in nm) and angles are reported.

Conformers	Cu		^{1}H **A** tensor				$\nu_{O\text{-}H}$
	g_z	$\|A_z\|$	a_{iso}	T_1	T_2	T_3	
OH opposite side of Al	2.295	341	-1.1	-12.1	-1.2	13.4	3658
OH same side of Al	2.280	371	-7.2	-13.6	-2.0	15.6	3650
Experimental	2.290	410	-2.0	-11.0	-2.5	13.5	3656*

*The experimental value of the $\nu_{O\text{-}H}$ is taken from Ref. 66.

Table S5. Computed spin-Hamiltonian parameters (at PBE0 with 40% of HF/CP(PPP) for ^{Cu}**A**- and **g**-tensors and DLPNO-CCSD/cc-pwCVQZ for H nuclei levels of theory) and anharmonic stretching frequencies of OH group bound to Cu (at B3LYP-D3(ABC)/pob-TZVP level of theory) for the two different conformations of [Cu^{II}(OH)(O-8MRs)$_3$] illustrated in Figure S7. Hyperfine couplings are reported in MHz whereas the stretching frequency is given in cm^{-1}.

The computed spin-Hamiltonian parameters and vibrational frequency of the O-H stretching for two possible conformations of [Cu^{II}(OH)(O-8MRs)$_3$] are reported in Table S5. The two conformers present computed EPR and vibrational properties in substantial agreement with the experiment, indicating that an average distribution of the two is likely present.

S7. Comparison of computed properties for a trigonal planar $[Cu^{II}(OH)(O\text{-}8MRs)_2$ and the four-coordinated $[Cu^{II}(OH)(O\text{-}8MRs)_3$ structure.

Framework bound $[Cu(OH)]^+$ species in zeolites are reported in literature as characterized by a trigonal planar geometry with the OH^- group hydrogen-bonded with an oxygen of the cage. To the best of our knowledge, this structure was first proposed by Andersen et al.[67] based on density functional theory calculations used to identity the Cu sites in dehydrated Cu-CHA. This structure is reported in Figure S8 along the structure we derive in this work and the computed spectroscopic observables are compared in Table S6.

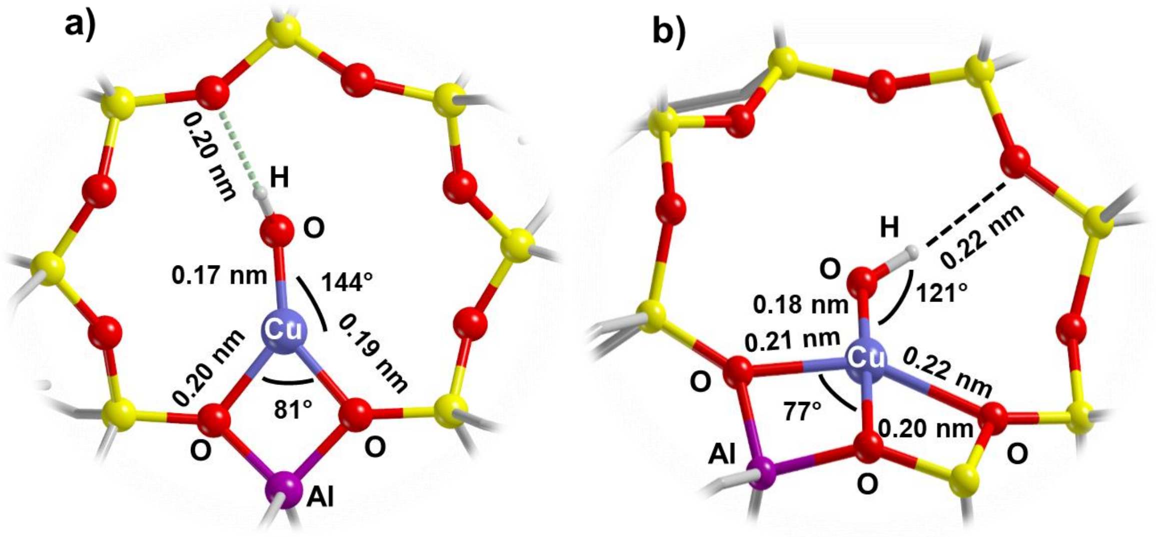

Figure S8 Possible structural geometries of framework bound $[Cu^{II}(OH)]^+$ species. a) three-coordinated $[Cu^{II}(OH)(O\text{-}8MRs)_2]$ structure; b) four-coordinated $[Cu^{II}(OH)(O\text{-}8MRs)_3]$ structure.

Inspection of Table S6 shows that even though the two structures have similar energies, the four-coordinated structure (Figure S8b) is the only one that reproduces quantitatively all spectroscopic parameters. In particular the ^1H hyperfine interaction provides a stringent constrain, the three-coordinated structure leading to computed values, which are off-set by an order of magnitude with respect to the experimental data.

The simulation of the experimental ^1H HYSCORE spectra performed using the data reported in Table S6 are reported in Figure S9 for the sake of comparison.

		$^{H}a_{iso}$ (MHz)	$^{H}T_{1}$ (MHz)	$^{H}T_{2}$ (MHz)	$^{H}T_{3}$ (MHz)	v_{OH} (cm^{-1})	ΔE (kJmol^{-1})
**Computed**	3C	-14.9	-16.8	-7.1	23.9	3626	+5
	4C	-1.1	-12.1	-1.2	13.4	3658	0
**Experimental**		-2.0	-11.0	-2.5	13.5	3656*	

*The experimental value of the $v_{O\text{-}H}$ is taken from Ref. 66.

Table S6 Computed ^{1}H hyperfine coupling constants and anharmonic stretching frequencies of OH group bound to Cu (v_{OH}) for [Cu^{II}(OH)(O-8MRs)$_2$] (3C) and [Cu^{II}(OH)(O-8MRs)$_3$] (4C) structures. ^{1}H hyperfine coupling constants were computed at DLPNO-CCSD/cc-pwCVQZ level of theory whereas the anharmonic stretching frequencies were computed at B3LYP-D3(ABC)/pob-TZVP level of theory.

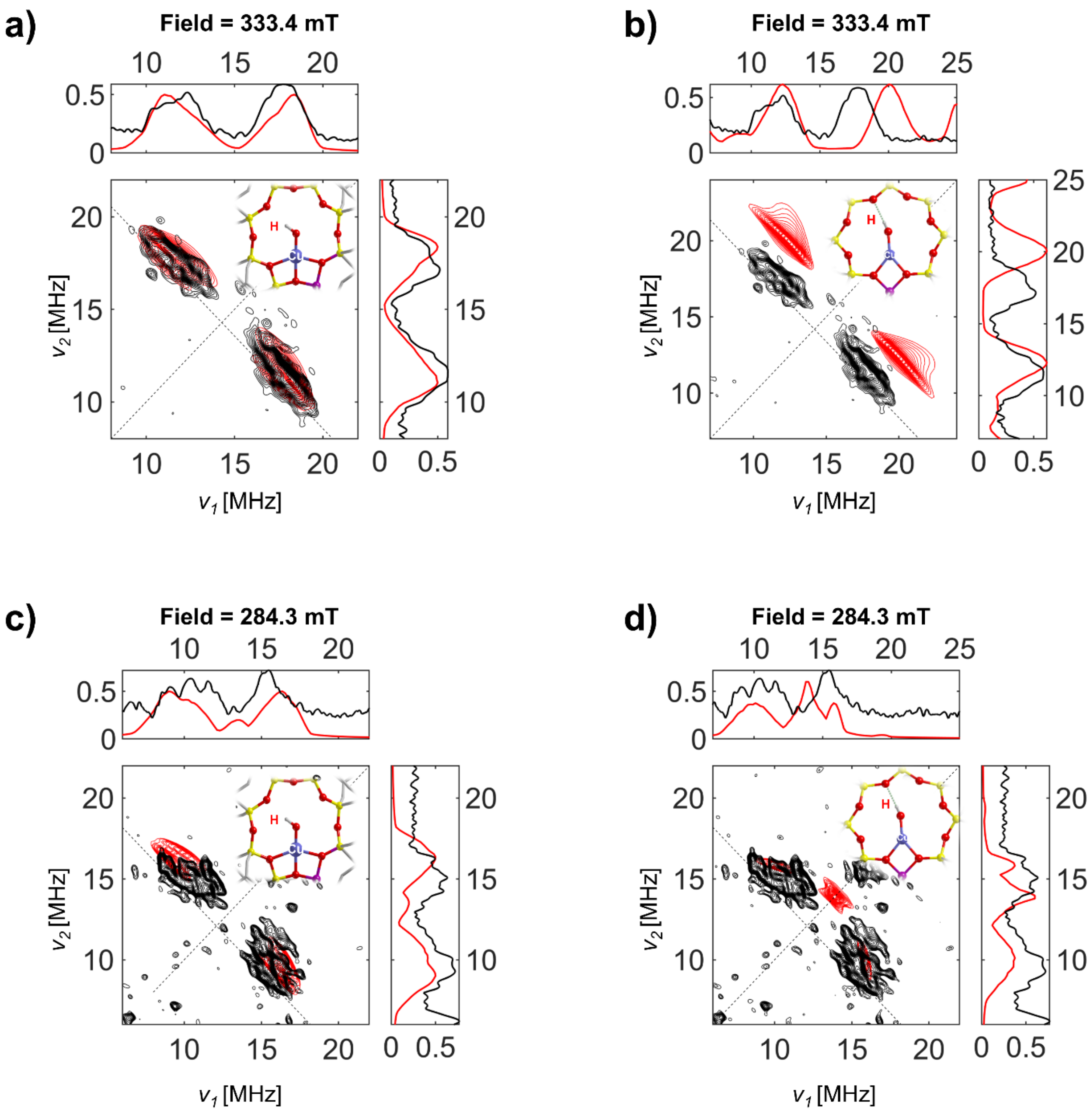

Figure S9 Experimental (black) and simulated (red) ^{1}H HYSCORE spectra taken at different magnetic field setting using the computed spin-Hamiltonian parameters reported in Table S6. a), c) four-coordinated structure; b, d) three-coordinated structure. The magnetic field settings are reported in the figure and correspond to the perpendicular (a,b) and the parallel component (c,d) of the spectrum.

S8. Simulations of ^{1}H HYSCORE spectrum of O_2-activated Cu-CHA(C) by using computed spin-Hamiltonian parameters

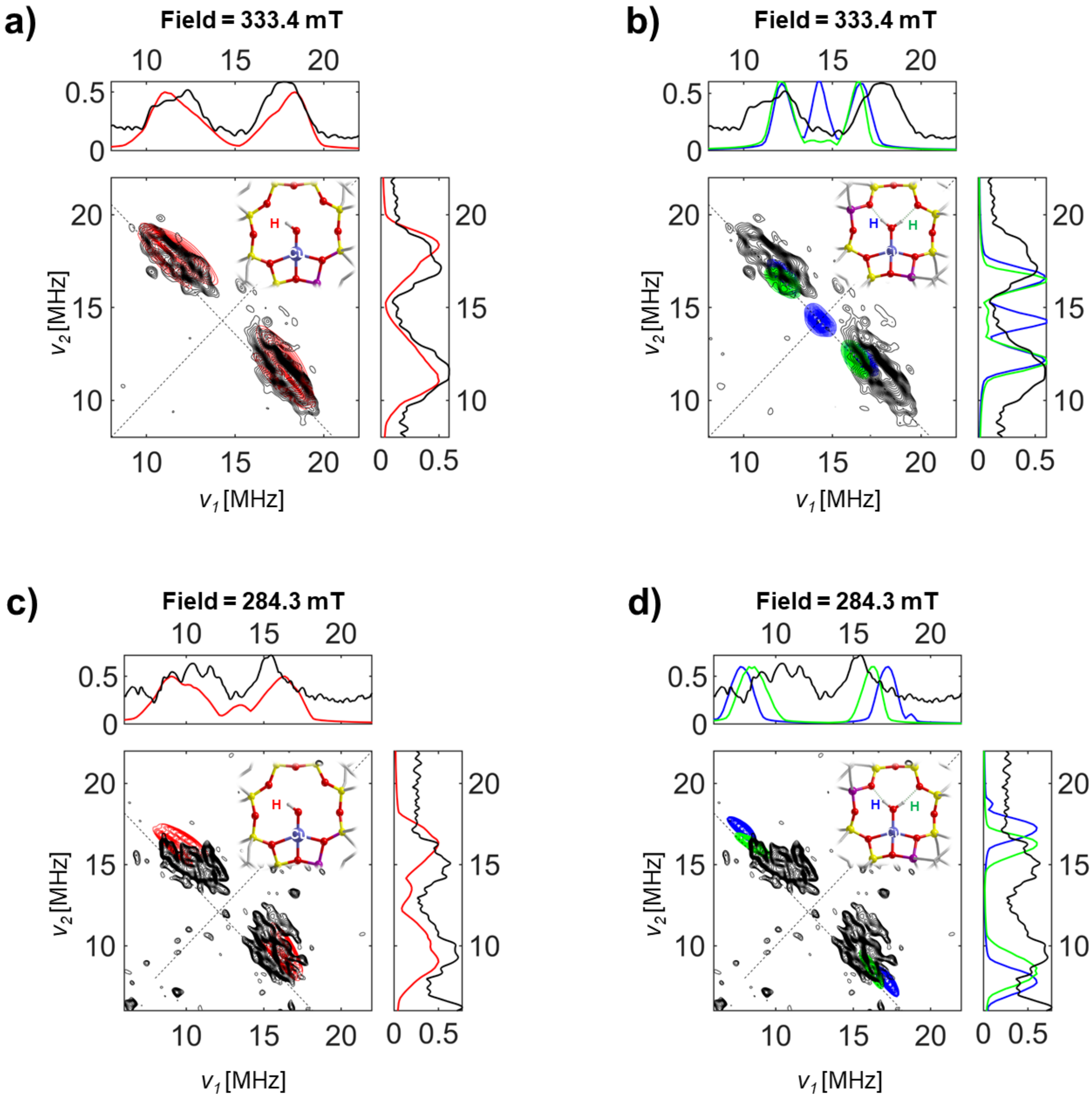

Figure S10. ^{1}H HYSCORE spectra (in black) of Cu-CHA(C) together with the computer simulation obtained at the reported magnetic fields by using computed spin-Hamiltonian parameters on a) and c) cluster model of [Cu^{II}OH(O-8MRs)$_3$]. The simulated proton signal is given in red; b) and d) cluster model of [Cu^{II}(H_2O)(O-8MRs)$_3$]. The simulated proton signals are given in green and blue. The relative sum projections are also

reported. The level of theory employed for the computed values was DSD-PBEP86/EPR-III for **A**-tensor of the H nuclei.

S9. References

(1) Fickel, D. W.; Lobo, R. F. Copper Coordination in Cu-SSZ-13 and Cu-SSZ-16 Investigated by Variable-Temperature XRD. *J. Phys. Chem. C* **2010**, *114* (3), 1633–1640.

(2) Zamadics, M.; Kevan, L. Electron Spin Resonance and Electron Spin Echo Modulation Studies of Cu(II) Ions in the Aluminosilicate Chabazite: A Comparison of Cu(II) Cation Location and Adsorbate Interaction with Isostructural Silicoaluminophosphate-34. *J. Phys. Chem.* **1992**, *96*, 8989–8993.

(3) Zones, S. I. U.S Patent, 4,544,538. 4,544,538, 1985.

(4) Höfer, P.; Grupp, A.; Nebenführ, H.; Mehring, M. Hyperfine Sublevel Correlation (Hyscore) Spectroscopy: A 2D ESR Investigation of the Squaric Acid Radical. *Chem. Phys. Lett.* **1986**, *132* (3), 279–282.

(5) Stoll, S.; Schweiger, A. EasySpin, a Comprehensive Software Package for Spectral Simulation and Analysis in EPR. *J. Magn. Reson.* **2006**, *178* (1), 42–55.

(6) Dovesi, R.; Erba, A.; Orlando, R.; Zicovich-Wilson, C. M.; Civalleri, B.; Maschio, L.; Rérat, M.; Casassa, S.; Baima, J.; Salustro, S.; Kirtman, B. Quantum-Mechanical Condensed Matter Simulations with CRYSTAL. *Wiley Interdiscip. Rev. Comput. Mol. Sci.* **2018**, *8* (4), e1360.

(7) Lee, C.; Yang, W.; Parr, R. G. Development of the Colle-Salvetti Correlation-Energy Formula into a Functional of the Electron Density. *Phys. Rev. B* **1988**, *37* (2), 785–789.

(8) Becke, A. D. Density-functional Thermochemistry. III. The Role of Exact Exchange. *J. Chem. Phys.* **1993**, *98* (7), 5648–5652.

(9) Fischer, M. Interaction of Water with (Silico)Aluminophosphate Zeotypes: A Comparative Investigation Using Dispersion-Corrected DFT. *Phys. Chem. Chem. Phys.* **2016**, *18* (23), 15738–15750.

(10) Signorile, M.; Damin, A.; Bonino, F.; Crocellà, V.; Lamberti, C.; Bordiga, S. The Role of Dispersive Forces Determining the Energetics of Adsorption in Ti Zeolites. *J. Comput. Chem.* **2016**, *37* (30), 2659–2666.

(11) Grimme, S.; Antony, J.; Ehrlich, S.; Krieg, H. A Consistent and Accurate Ab Initio Parametrization of Density Functional Dispersion Correction (DFT-D) for the 94 Elements H-Pu. *J. Chem. Phys.* **2010**, *132* (15), 154104.

(12) Grimme, S.; Ehrlich, S.; Goerigk, L. Effect of the Damping Function in Dispersion Corrected Density Functional Theory. *J. Comput. Chem.* **2011**, *32* (7), 1456–1465.

(13) Peintinger, M. F.; Oliveira, D. V.; Bredow, T. Consistent Gaussian Basis Sets of Triple-Zeta Valence with Polarization Quality for Solid-State Calculations. *J. Comput. Chem.* **2013**, *34* (6), 451–459.

(14) Eichkorn, K.; Weigend, F.; Treutler, O.; Ahlrichs, R. Auxiliary Basis Sets for Main Row Atoms and Transition Metals and Their Use to Approximate Coulomb Potentials. *Theor. Chem. Acc.* **1997**, *97* (1–4), 119–124.

(15) Zicovich-Wilson, C. M.; Pascale, F.; Roetti, C.; Saunders, V. R.; Orlando, R.; Dovesi, R. Calculation of the Vibration Frequencies of α-Quartz: The Effect of Hamiltonian and Basis Set. *J. Comput. Chem.* **2004**, *25* (15), 1873–1881.

(16) Pascale, F.; Zicovich-Wilson, C. M.; López Gejo, F.; Civalleri, B.; Orlando, R.; Dovesi, R. The Calculation of the Vibrational Frequencies of Crystalline Compounds and Its Implementation in the CRYSTAL Code. *J. Comput. Chem.* **2004**, *25* (6), 888–897.

(17) Carteret, C.; De La Pierre, M.; Dossot, M.; Pascale, F.; Erba, A.; Dovesi, R. The Vibrational Spectrum of $CaCO_3$ Aragonite: A Combined Experimental and Quantum-Mechanical Investigation. *J. Chem. Phys.* **2013**, *138* (1), 14201.

(18) Ugliengo, P.; Pascale, F.; Mérawa, M.; Labéguerie, P.; Tosoni, S.; Dovesi, R. Infrared Spectra of Hydrogen-Bonded Ionic Crystals: Ab Initio Study of $Mg(OH)_2$ and β-$Be(OH)_2$. *J. Phys. Chem. B* **2004**, *108* (36), 13632–13637.

(19) Pascale, F.; Tosoni, S.; Zicovich-Wilson, C.; Ugliengo, P.; Orlando, R.; Dovesi, R. Vibrational Spectrum of Brucite, $Mg(OH)_2$: A Periodic Ab Initio Quantum Mechanical Calculation Including OH Anharmonicity. *Chem. Phys. Lett.* **2004**, *396* (4–6), 308–315.

(20) Lindberg, B. A New Efficient Method for Calculation of Energy Eigenvalues and Eigenstates of the One-dimensional Schrödinger Equation. *J. Chem. Phys.* **1998**, *88* (6), 3805.

(21) Neese, F. Software Update: The ORCA Program System, Version 4.0. *WIREs Comput. Mol. Sci.* **2018**, *8* (1), e1327.

(22) Neese, F. Software Update: The ORCA Program System—Version 5.0. *Wiley Interdiscip. Rev. Comput. Mol. Sci.* **2022**, e1606.

(23) Remenyi, C.; Reviakine, R.; Arbuznikov, A. V.; Vaara, J.; Kaupp, M. Spin-Orbit Effects on Hyperfine Coupling Tensors in Transition Metal Complexes Using Hybrid Density Functionals and Accurate Spin-Orbit Operators. *J. Phys. Chem. A* **2004**, *108* (23), 5026–5033.

(24) Heß, B. A.; Marian, C. M.; Wahlgren, U.; Gropen, O. A Mean-Field Spin-Orbit Method Applicable to Correlated Wavefunctions. *Chem. Phys. Lett.* **1996**, *251* (5–6), 365–371.

(25) Sinnecker, S.; Slep, L. D.; Bill, E.; Neese, F. Performance of Nonrelativistic and Quasi-Relativistic Hybrid DFT for the Prediction of Electric and Magnetic Hyperfine Parameters in ^{57}Fe Mössbauer Spectra. *Inorg. Chem.* **2005**, *44* (7), 2245–2254.

(26) Weigend, F.; Ahlrichs, R. Balanced Basis Sets of Split Valence, Triple Zeta Valence and Quadruple Zeta Valence Quality for H to Rn: Design and Assessment of Accuracy. *Phys. Chem. Chem. Phys.* **2005**, *7* (18), 3297–3305.

(27) Barone, V. Structure, Thermochemistry, and Magnetic Properties of Binary Copper Carbonyls by a Density-Functional Approach. **1995**, *99*, 11659–11666.

(28) Saitow, M.; Neese, F. Accurate Spin-Densities Based on the Domain-Based Local Pair-Natural Orbital Coupled-Cluster Theory. *J. Chem. Phys.* **2018**, *149* (3), 034104.

(29) Riplinger, C.; Neese, F. An Efficient and near Linear Scaling Pair Natural Orbital Based Local Coupled Cluster Method. *J. Chem. Phys.* **2013**, *138* (3), 034106.

(30) Saitow, M.; Becker, U.; Riplinger, C.; Valeev, E. F.; Neese, F. A New Near-Linear Scaling, Efficient and Accurate, Open-Shell Domain-Based Local Pair Natural Orbital Coupled Cluster Singles and Doubles Theory. *J. Chem. Phys.* **2017**, *146* (16), 164105.

(31) Dunning, T. H. Gaussian Basis Sets for Use in Correlated Molecular Calculations. I. The Atoms Boron through Neon and Hydrogen. *J. Chem. Phys.* **1989**, *90* (2), 1007–1023.

(32) Peterson, K. A.; Dunning, T. H. Accurate Correlation Consistent Basis Sets for Molecular Core–Valence Correlation Effects: The Second Row Atoms Al–Ar, and the First Row Atoms B–Ne Revisited. *J. Chem. Phys.* **2002**, *117* (23), 10548–10560.

(33) Reiher, M.; Wolf, A. Exact Decoupling of the Dirac Hamiltonian. I. General Theory. *J. Chem. Phys.*

2004, *121* (5), 2037.

(34) Reiher, M.; Wolf, A. Exact Decoupling of the Dirac Hamiltonian. II. The Generalized Douglas–Kroll–Hess Transformation up to Arbitrary Order. *J. Chem. Phys.* **2004**, *121* (22), 10945.

(35) Wolf, A.; Reiher, M. Exact Decoupling of the Dirac Hamiltonian. III. Molecular Properties. *J. Chem. Phys.* **2006**, *124* (6), 064102.

(36) Malkin, E.; Malkin, I.; Malkina, O. L.; Malkin, V. G.; Kaupp, M. Scalar Relativistic Calculations of Hyperfine Coupling Tensors Using the Douglas–Kroll–Hess Method with a Finite-Size Nucleus Model. *Phys. Chem. Chem. Phys.* **2006**, *8* (35), 4079–4085.

(37) Mastalerz, R.; Lindh, R.; Reiher, M. The Douglas–Kroll–Hess Electron Density at an Atomic Nucleus. *Chem. Phys. Lett.* **2008**, *465* (1–3), 157–164.

(38) Sandhoefer, B.; Kossmann, S.; Neese, F. Derivation and Assessment of Relativistic Hyperfine-Coupling Tensors on the Basis of Orbital-Optimized Second-Order Møller–Plesset Perturbation Theory and the Second-Order Douglas–Kroll–Hess Transformation. *J. Chem. Phys.* **2013**, *138* (10), 104102.

(39) Carl, P. J.; Larsen, S. C. Variable-Temperature Electron Paramagnetic Resonance Studies of Copper-Exchanged Zeolites. *J. Catal.* **1999**, *182* (1), 208–218.

(40) Carl, P. J.; Larsen, S. C. EPR Study of Copper-Exchanged Zeolites: Effects of Correlated g- and A-Strain, Si/Al Ratio, and Parent Zeolite. *J. Phys. Chem. B* **2000**, *104* (28), 6568–6575.

(41) Peisach, J.; Blumberg, W. E. Structural Implications Derived from the Analysis of Electron Paramagnetic Resonance Spectra of Natural and Artificial Copper Proteins. *Arch. Biochem. Biophys.* **1974**, *165* (2), 691–708.

(42) Bruzzese, P. C.; Salvadori, E.; Jäger, S.; Hartmann, M.; Civalleri, B.; Pöppl, A.; Chiesa, M. ^{17}O-EPR Determination of the Structure and Dynamics of Copper Single-Metal Sites in Zeolites. *Nat. Commun.* **2021**, *12* (1), 4638.

(43) Hofer, P.; Eichhoff, U. 75 Years of EPR: Milestones in the 62 Years Bruker EPR History. In *Magnetic Resonance and its Applications*; 2020; pp 41–43.

(44) Pappas, D. K.; Borfecchia, E.; Dyballa, M.; Pankin, I. A.; Lomachenko, K. A.; Martini, A.; Signorile, M.; Teketel, S.; Arstad, B.; Berlier, G.; Lamberti, C.; Bordiga, S.; Olsbye, U.; Lillerud, K. P.; Svelle, S.; Beato, P. Methane to Methanol: Structure-Activity Relationships for Cu-CHA. *J. Am. Chem. Soc.* **2017**, *139* (42), 14961–14975.

(45) Paolucci, C.; Khurana, I.; Parekh, A. A.; Li, S.; Shih, A. J.; Li, H.; Di Iorio, J. R.; Albarracin-Caballero, J. D.; Yezerets, A.; Miller, J. T.; Delgass, W. N.; Ribeiro, F. H.; Schneider, W. F.; Gounder, R. Dynamic Multinuclear Sites Formed by Mobilized Copper Ions in NO_x Selective Catalytic Reduction. *Science* **2017**, *357*, 898–903.

(46) Paolucci, C.; Parekh, A. A.; Khurana, I.; Di Iorio, J. R.; Li, H.; Albarracin Caballero, J. D.; Shih, A. J.; Anggara, T.; Delgass, W. N.; Miller, J. T.; Ribeiro, F. H.; Gounder, R.; Schneider, W. F. Catalysis in a Cage: Condition-Dependent Speciation and Dynamics of Exchanged Cu Cations in SSZ-13 Zeolites. *J. Am. Chem. Soc.* **2016**, *138* (18), 6028–6048.

(47) Song, J.; Wang, Y.; Walter, E. D.; Washton, N. M.; Mei, D.; Kovarik, L.; Engelhard, M. H.; Prodinger, S.; Wang, Y.; Peden, C. H. F.; Gao, F. Toward Rational Design of Cu/SSZ-13 Selective Catalytic Reduction Catalysts: Implications from Atomic-Level Understanding of Hydrothermal Stability. *ACS Catal.* **2017**, *7* (12), 8214–8227.

(48) Zhang, Y.; Wu, Y.; Peng, Y.; Li, J.; Walter, E. D.; Chen, Y.; Washton, N. M.; Szanyi, J.; Wang, Y.; Gao, F. Quantitative Cu Counting Methodologies for Cu/SSZ-13 Selective Catalytic Reduction Catalysts by Electron Paramagnetic Resonance Spectroscopy. *J. Phys. Chem. C* **2020**, *124* (51),

28061–28073.

(49) Villamaina, R.; Liu, S.; Nova, I.; Tronconi, E.; Ruggeri, M. P.; Collier, J.; York, A.; Thompsett, D. Speciation of Cu Cations in Cu-CHA Catalysts for NH_3-SCR: Effects of SiO_2/AlO_3 Ratio and Cu-Loading Investigated by Transient Response Methods. *ACS Catal.* **2019**, *9* (10), 8916–8927.

(50) Godiksen, A.; Stappen, F. N.; Vennestrøm, P. N. R.; Giordanino, F.; Rasmussen, S. B.; Lundegaard, L. F.; Mossin, S. Coordination Environment of Copper Sites in Cu-CHA Zeolite Investigated by Electron Paramagnetic Resonance. *J. Phys. Chem. C* **2014**, *118* (40), 23126–23138.

(51) Villamaina, R.; Gramigni, F.; Iacobone, U.; Liu, S.; Nova, I.; Tronconi, E.; Ruggeri, M. P.; Collier, J.; York, A. P. E.; Thompsett, D. The H_2O Effect on Cu Speciation in Cu-CHA-Catalysts for NH_3-SCR Probed by NH_3 Titration. *Catalysts,* **2021**, *11(7)*, *759*.

(52) Sushkevich, V. L.; Smirnov, A. V.; Van Bokhoven, J. A. Autoreduction of Copper in Zeolites: Role of Topology, Si/Al Ratio, and Copper Loading. *J. Phys. Chem. C* **2019**, *123* (15), 9926–9934.

(53) Sushkevich, V. L.; Van Bokhoven, J. A. Revisiting Copper Reduction in Zeolites: The Impact of Autoreduction and Sample Synthesis Procedure. *Chem. Commun.* **2018**, *54* (54), 7447–7450.

(54) Trout, B. L.; Chakraborty, A. K.; Bell, A. T. Local Spin Density Functional Theory Study of Copper Ion-Exchanged ZSM-5. *J. Phys. Chem.* **1996**, *100* (10), 4173–4179.

(55) Boguslawski, K.; Marti, K. H.; Legeza, Ö.; Reiher, M. Accurate Ab Initio Spin Densities. *J. Chem. Theory Comput.* **2012**, *8* (6), 1970–1982.

(56) Improta, R.; Barone, V. Interplay of Electronic, Environmental, and Vibrational Effects in Determining the Hyperfine Coupling Constants of Organic Free Radicals. *Chem. Rev.* **2004**, *104* (3), 1231–1254.

(57) Neese, F. Prediction of Molecular Properties and Molecular Spectroscopy with Density Functional Theory: From Fundamental Theory to Exchange-Coupling. *Coordination Chemistry Reviews*. Elsevier March 1, 2009, pp 526–563.

(58) Kossmann, S.; Neese, F. Correlated Ab Initio Spin Densities for Larger Molecules: Orbital-Optimized Spin-Component-Scaled MP2 Method. *J. Phys. Chem. A* **2010**, *114* (43), 11768–11781.

(59) Perdew, J. P.; Zunger, A. Self-Interaction Correction to Density-Functional Approximations for Many-Electron Systems. *Phys. Rev. B* **1981**, *23* (10), 5048–5079.

(60) Grimme, S. Semiempirical GGA-Type Density Functional Constructed with a Long-Range Dispersion Correction. *J. Comput. Chem.* **2006**, *27* (15), 1787–1799.

(61) Kossmann, S.; Kirchner, B.; Neese, F. Performance of Modern Density Functional Theory for the Prediction of Hyperfine Structure: Meta-GGA and Double Hybrid Functionals. *Mol. Phys.* **2007**, *105* (15–16), 2049–2071.

(62) Tran, V. A.; Neese, F. Double-Hybrid Density Functional Theory for g-Tensor Calculations Using Gauge Including Atomic Orbitals. *J. Chem. Phys.* **2020**, *153* (5), 54105.

(63) Gómez-Piñeiro, R. J.; Pantazis, D. A.; Orio, M. Comparison of Density Functional and Correlated Wave Function Methods for the Prediction of Cu(II) Hyperfine Coupling Constants. *ChemPhysChem* **2020**, *21* (24), 2667–2679.

(64) Witwicki, M.; Walencik, P. K.; Jezierska, J. How Accurate Is Density Functional Theory in Predicting Spin Density? An Insight from the Prediction of Hyperfine Coupling Constants. *J. Mol. Model.* **2019**, *26* (1), 10.

(65) Goerigk, L.; Hansen, A.; Bauer, C.; Ehrlich, S.; Najibi, A.; Grimme, S. A Look at the Density Functional Theory Zoo with the Advanced GMTKN55 Database for General Main Group Thermochemistry, Kinetics and Noncovalent Interactions. *Phys. Chem. Chem. Phys.* **2017**, *19* (48), 32184–32215.

(66) Borfecchia, E.; Lomachenko, K. A.; Giordanino, F.; Falsig, H.; Beato, P.; Soldatov, A. V.; Bordiga, S.; Lamberti, C. Revisiting the Nature of Cu Sites in the Activated Cu-SSZ-13 Catalyst for SCR Reaction. *Chem. Sci.* **2014**, *6* (1), 548–563.

(67) Andersen, C. W.; Bremholm, M.; Vennestrøm, P. N. R.; Blichfeld, A. B.; Lundegaard, L. F.; Iversen, B. B. Location of Cu^{2+} in CHA Zeolite Investigated by X-Ray Diffraction Using the Rietveld/Maximum Entropy Method. *IUCrJ* **2014**, *1* (6), 382–386.
